# UK Iatrogenic Creutzfeldt–Jakob disease: investigating human prion transmission across genotypic barriers using human tissue-based and molecular approaches

**DOI:** 10.1007/s00401-016-1638-x

**Published:** 2016-11-03

**Authors:** Diane L. Ritchie, Marcelo A. Barria, Alexander H. Peden, Helen M. Yull, James Kirkpatrick, Peter Adlard, James W. Ironside, Mark W. Head

**Affiliations:** 1 National CJD Research & Surveillance Unit, Centre for Clinical Brain Sciences, Deanery of Clinical Sciences, The University of Edinburgh, Edinburgh, UK; 2 University College London Institute of Child Health, 30 Guilford Street, London, WC1N 1EH UK; 3 National CJD Research & Surveillance Unit, University of Edinburgh, Bryan Matthews Building, Western General Hospital, Edinburgh, EH4 2XU UK

**Keywords:** Creutzfeldt–Jakob disease, Prion, Iatrogenic, Growth hormone, Disease phenotype, Agent strain

## Abstract

Creutzfeldt–Jakob disease (CJD) is the prototypic human prion disease that occurs most commonly in sporadic and genetic forms, but it is also transmissible and can be acquired through medical procedures, resulting in iatrogenic CJD (iCJD). The largest numbers of iCJD cases that have occurred worldwide have resulted from contaminated cadaveric pituitary-derived human growth hormone (hGH) and its use to treat primary and secondary growth hormone deficiency. We report a comprehensive, tissue-based and molecular genetic analysis of the largest series of UK hGH-iCJD cases reported to date, including in vitro kinetic molecular modelling of genotypic factors influencing prion transmission. The results show the interplay of prion strain and host genotype in governing the molecular, pathological and temporal characteristics of the UK hGH-iCJD epidemic and provide insights into the adaptive mechanisms involved when prions cross genotypic barriers. We conclude that all of the available evidence is consistent with the hypothesis that the UK hGH-iCJD epidemic resulted from transmission of the V2 human prion strain, which is associated with the second most common form of sporadic CJD.

**Electronic supplementary material:**

The online version of this article (doi:10.1007/s00401-016-1638-x) contains supplementary material, which is available to authorized users.

## Introduction

Prion diseases are a group of fatal transmissible neurodegenerative disorders that occur naturally in man and a range of other species [12]. All are associated with the conversion of a normal host cellular protein, the prion protein (PrP^C^), into a misfolded disease-associated form, PrP^Sc^, which accumulates in the central nervous system (CNS) [29]. The prion hypothesis states that PrP^Sc^ is the principal (if not the sole) component of the transmissible agents responsible for this group of diseases [30]; this hypothesis is supported by an increasing body of experimental evidence [9]. However, the precise relationship between the conformation of PrP^Sc^ in different forms of prion disease and the biological properties of the transmissible agents (strain characteristics) in different prion diseases is still unresolved.

Unlike the more common forms of human neurodegenerative diseases, human prion diseases occur in sporadic, genetic and acquired forms [12]. The acquired human prion diseases that have resulted from human-to-human transmission include kuru (now considered to be extinct) and iatrogenic Creutzfeldt-Jakob disease (iCJD), with variant Creutzfeldt–Jakob disease (vCJD) resulting from bovine to human transmission of the bovine spongiform encephalopathy (BSE) agent. Iatrogenic transmission of Creutzfeldt–Jakob disease (CJD) has occurred in many countries across the world as a consequence of transmission via contaminated neurosurgical instruments, intracerebral electroencephalography needles, corneal transplantation, human dura mater grafts, inoculation with human pituitary hormones and (in the case of vCJD) transfusion of packed red blood cells [14]. The vast majority of iCJD cases have occurred in human dura mater (hDM) graft recipients and in recipients of human growth hormone (hGH) derived from cadaveric pituitaries [5]. The incubation periods in the worldwide cohort of iCJD cases are markedly variable, with the shortest incubation periods occurring in those exposed via surgical instruments or electrodes used on the CNS (1–2.3 years) and the longest occurring in hDM graft recipients (1.3–30 years), and human pituitary-derived hGH and gonadotrophin recipients (5–42 years) [5]. Estimation of incubation periods in human pituitary hormone recipients is difficult, since the patients are often treated over a period of years; the time period from the mid-point of pituitary hormone treatment to the onset of clinical symptoms of iCJD is often used as an estimate for the incubation period [33].

The use of pituitary-derived hGH in the treatment of primary and secondary growth hormone deficiency in the United Kingdom (UK) began as a clinical trial in 1959 and became a centrally administered National Health Service activity in 1976 [33]. In 1985, the first cases of iCJD in patients treated with pituitary-derived hGH were reported in the United States of America (USA) and in the UK [10, 28]. Consequently, the use of pituitary-derived hGH for the treatment of growth hormone deficiency was abandoned in the UK, USA and many other countries and biosynthetic growth hormone is now used for this purpose. The presence of prion infectivity in hGH preparations in the USA was confirmed by experimental intracerebral inoculation of samples of pituitary-derived hGH into primates, which resulted in the onset of neurological symptoms in a squirrel monkey after an incubation period of over 5.5 years [11]. Post-mortem examination of the brain of this animal confirmed the presence of a subacute spongiform encephalopathy.

In the UK, 1849 patients were treated with hGH in the national scheme from 1959 until 1985 [33]. Since 1985, 78 deaths from iatrogenic CJD have occurred in this cohort, and it has been identified that one particular preparation of UK pituitary-derived hGH (the Hartree-modified Wilhelmi preparation) had been administered to all hGH recipients who had developed iCJD [31, 33]. It has also been estimated that the risk of developing iCJD in the UK hGH recipient cohort was greatest in those patients who received treatment at ages 8-10 years with a peak incubation period of 20 years [33]. Estimated incubation periods in the UK hGH-iCJD patients range from 7 to 40 years [31]; these prolonged incubation periods are reminiscent of those occurring in kuru, where incubation periods of over 40 years have been reported [8]. In contrast, only eight cases of iCJD in hDM graft recipients (hDM-iCJD) have been reported in the UK, with incubation periods ranging from 3.8 to 14.8 years [13].

Incubation periods in prion diseases have been studied extensively under experimental conditions and appear to be significantly influenced by both recipient (host) genetic factors and the strain properties of the infectious agent [12]. The principal human genetic factor influencing this incubation period is the codon 129 polymorphism in the prion protein gene (*PRNP*) of the recipient. It has long been recognised that homozygosity at this locus may predispose to both iCJD and sporadic CJD (sCJD), but differences in the frequency of the three possible genotypes (MM, MV and VV) have been reported in hGH-iCJD cohorts in different countries; in particular, most cases of hGH-iCJD in France have belonged to the MM subgroup, whilst in the UK the VV and MV subgroups predominate [4]. These differences may also reflect the nature of the strain(s) of CJD (presumably sCJD) that were responsible for the iatrogenic disease transmission.

Despite the fact that iCJD in hGH recipients has occurred in over 200 patients across the world, there have been very few detailed studies of the neuropathology, prion protein biochemistry and prion protein seeding activity in this form of human prion disease. Most reports describe only small numbers of cases that are not necessarily representative of the cohort in question, particularly with respect to the year of diagnosis in relation to the evolving time period since iatrogenic exposure to contaminated hGH ceased. We now report a detailed study of the largest cohort of hGH-iCJD cases reported to date, with detailed characterisation of PrP^Sc^ in the brain, correlation with genetic and pathological factors and an exploration of the in vitro PrP seeding activity present in different genetic subgroups. These cases are compared with a limited number of cases of hDM-iCJD in the UK, but also a very much larger group of sCJD cases drawn from the UK population over a similar time period. The detailed examination of these cohorts has allowed us a unique opportunity to ask fundamental questions about person-to-person transmission of prion disease and the molecular mechanisms involved.

## Materials and methods

### Cases, inclusion criteria and tissue specimens

All CJD cases examined were of UK origin and were referred to the National CJD Research and Surveillance Unit (NCJDRSU) between 1991 and 2015. Diagnoses were made according to WHO criteria [35]. Inclusion criteria for biochemical investigation of iCJD cases were the availability of frozen CNS tissue taken at post-mortem, appropriate consent and ethical approval for retention and research use and the availability of basic clinical data. All patients referred to the NCJDRSU with a diagnosis of CJD who had received hGH therapy or a hDM graft and met these criteria were included in the study. The study identification number and basic patient demographic data for 21 hGH-iCJD and three hDM-iCJD cases are detailed in Online Resource Table 1. Where available, samples of both frozen grey matter and the corresponding fixed samples from the cerebral cortex (frontal, parietal, temporal and occipital cortex), cerebellum, thalamus and spinal cord were examined. Data from a larger subset of the UK hGH-iCJD cases, including some of those for which frozen tissues were not available were included in genetic analyses. Data from 1080 unselected cases of UK sCJD of known *PRNP* codon 129, with deaths between 1990 and 2015 were used for genetic comparison with the UK hGH-iCJD cases. A smaller group of UK sCJD cases (*n* = 108) that were referred to the NCJDRSU during the period 1990–2011 were used for biochemical comparison with the hGH-iCJD group. These cases were selected on the basis of consent for research use, availability of clinical information and sufficient frozen tissue available (minimal set comprising temporal cortex, occipital cortex, parietal cortex and thalamus) for PrP^res^ type classification according to the method of Parchi et al. [23]. All CJD tissue samples were provided by request from the NCJDRSU Brain and Tissue Bank in Edinburgh, UK, which is part of the MRC Edinburgh Brain Bank.

### Neuropathological analysis

Formalin fixed, formic acid treated, paraffin embedded tissue samples from the frontal, parietal, occipital and temporal cortices, hippocampus, amygdala, basal ganglia, thalamus, brainstem, cerebellum and spinal cord (where available) were stained with haematoxylin and eosin (H&E) and immunohistochemistry carried out for the prion protein. Immunohistochemistry was performed using two monoclonal antibodies recognising the prion protein; 12F10/epitope: amino acids 142–160 (Bioquote Ltd, York, UK) and KG9/epitope: amino acids 140–160 (TSE Resource Centre, Roslin Institute, UK) in combination with the highly sensitive Novolink™ Polymer Detection System (Leica Biosystems, UK; Product RE7280-K). Briefly, 5 µm paraffin embedded tissue sections were autoclaved in distilled water (dH_2_O) at 121 °C for 10 min followed by immersion in 96% formic acid for 5 min. Tissue sections were thoroughly washed in water before incubating in proteinase K solution (5 µg/ml) for 5 min. Sections were washed in Tris-buffered saline (TBS) [50 mM Tris; 150 mM NaCl; pH 7.6] before incubating with the primary antibodies (12F10, 30 ng/ml; KG9, 40 ng/ml) diluted in antibody diluent (Leica Biosystems, UK: Product RE7133-CE) for 1 h at room temperature. After 3 × 5 min washes in TBS, immunolabeling was completed using the Novolink™ Polymer Detection System. After a final wash, staining was visualised with 3, 3′-diaminobenzidine (DAB).

Tissue sections were analysed independently by two experienced assessors (DLR and JWI). The distribution, severity and nature of the spongiform change, neuronal loss, gliosis and amyloid plaque formation were assessed on H&E sections and scored in a semiquantitative manner (0-absent, 1-mild, 2-moderate, 3-severe) [23]. The distribution, severity and nature of the abnormal PrP accumulation detected by immunohistochemistry were also recorded [12]. This allowed subclassification by histotyping [23, 25] to determine whether the range of neuropathological phenotypes identified in the iCJD patients corresponded to those occurring in the recognised sCJD subtypes, or not.

### Western blot analysis and classification of PrP^res^ types

PrP^res^ types present in brain samples from the hGH-iCJD, hDM-iCJD and sCJD cases were determined using the refined Western blot protocol of Parchi et al. [23]. Briefly, 100 mg samples of frozen CNS samples were subjected to stringent sample preparation (including strong buffering to pH6.9 at 37 °C) and digestion with proteinase K (Roche, Lewes, UK) using 10U/ml corresponding to ~200 µg/ml (depending on the batch) at 37 °C for 1 h prior to Western blot analysis. Immunodetection of PrP was carried out using monoclonal antibody 3F4 (Millipore, Watford, UK; Catalogue number MAB1564) at a final concentration of 75 ng/mL for 1 h, or the monoclonal antibody 12B2 (Central Veterinary Institute of Wageningen UR, Lelystad, The Netherlands) at a final concentration of 200 ng/ml for 1 h. This protocol allows a distinction to be made between PrP^res^ types based on the migration of the unglycosylated (bottom) band of the typical triplet glycoform pattern. Three different unglycosylated PrP^res^ fragment mobilities are recognised within the 21–19 kDa range: type 1 in which the unglycosylated PrP^res^ has a molecular mass of ~21 kDa, type 2 in which the unglycosylated PrP^res^ fragment has a molecular mass of ~19 kDa, and an additional type with a mobility of ~20 kDa migrating between type 1 and 2, and therefore referred to as type i (for intermediate). PrP^res^ types 1, i and 2 are not mutually exclusive. Certain individual samples and cases contain more than one PrP^res^ type. This diversity is recognised by classifying such samples and cases as 1 + 2 or i + 2.

### Real-time quaking induced conversion (RT-QuIC)

The method used for the RT-QuIC in vitro conversion assay was the one used previously [26, 27] with minor modifications. Full length hamster recombinant PrP (recPrP, aa 23–231; GenBank accession no. K02234) was used as a substrate for seeded conversion. The hamster *Prnp* sequence encodes methionine at the position equivalent to codon 129 of the human *PRNP* gene. Brain samples to seed the reactions were prepared by homogenizing frozen, pre-weighed cerebral cortex tissue samples in phosphate buffered saline (PBS) containing 1 mM EDTA, 150 mM NaCl, 0.5% Triton X-100 and Complete™ Mini EDTA-free Protease Inhibitor Cocktail (Roche). The tissue was disrupted using one cycle of lysis (45 s, 6 ms^−1^) in a FastPrep-24 homogeniser (MP Biomedicals, Santa Ana, CA, USA). An appropriate volume of homogenization buffer was used to give a final tissue concentration of 10% (w/v). Cellular debris was cleared from the homogenates by centrifugation at 5200* g* at 4 °C for 5 m. Further serial tenfold dilutions of the cleared tissue homogenate were made using PBS. The 100 ml RT-QuIC reactions were set up in quadruplicate in the wells of a clear-bottomed black 96-well microplate (Fisher Scientific, Loughborough, UK). Stock solutions containing the reaction components were filtered through a 0.22 μM Millex PES filter prior to making a master mix. recPrP was the final addition to the master mix, which was mixed by gently inverting the tube and then dispensed into the wells in 98 μl volumes. At least four replicate analyses were performed on each patient sample. The RT-QuIC reactions (final volume = 100 μl, recPrP final concentration = 0.1 mg/ml) were initiated by the addition of 2 μl of a 10^3^-fold dilution of the 10% (w/v) homogenate, which is equivalent to 2 × 10^−7^ g brain (wet mass) per 100 μl reaction. After adding this volume of diluted brain homogenate, the final reactions contained PBS, 324 mM NaCl (including the NaCl present in PBS), 1 mM EDTA, 10 mM ThT, 1 × 10^-7^% Triton™ X-100 and 0.1 mg/ml recPrP. The plates were sealed with film, incubated at 42 °C and shaken intermittently (87 s shaking at 900 rpm in a double orbital configuration followed by a 33 s rest) using a FLUOstar OMEGA microplate reader (BMG Labtech). Fluorescence readings were taken at 480 nm every 15 min from the bottom of the wells after excitation with 20 flashes per well at 450 nm. To quantify prion seeding efficiency we used a method based on the one described by Shi et al. [32]. In this method, the lag time to conversion is used as an inverse measure of seeding potential. The lag period to the start of conversion was taken to be the time (in hours) from the start of the assay to when the RT-QuIC fluorescence reading was three times the average reading at 0.5 h (the baseline). A mean lag period for four replicate analyses was thus obtained for each patient.

### Protein misfolding cyclic amplification

Normal brain homogenates, from humanised transgenic mice of the *PRNP* codon 129 methionine homozygous (129MM) and valine homozygous (129VV) genotypes, were prepared using a manual homogenizer and conversion buffer (Phosphate-Buffered Saline 1X, 150 mM NaCl, 1% Triton X-100 and 1X protease inhibitors) to obtain a final 10% weight/volume solution (termed the PMCA “substrate”). The homogenized tissue was cleared by centrifugation at 2000 rpm for 40 s in a refrigerated centrifuge (4 °C), and the supernatant was aliquoted and stored at −80 °C. The CJD brain homogenates (termed PMCA “seeds”) were prepared following the same method used for substrate preparation. PMCA experiments were carried out as described previously [1]. Briefly, aliquots of 10% brain homogenate substrate were mixed with 10% CJD brain homogenates seeds in PCR tubes. Low molecular weight heparin was included at 100 μg/ml for all PMCA reactions. Prior to the amplification procedure, 19 μL of the PMCA reaction mixture were taken (from the final volume of 120 μl) for each reaction (referred to as the “frozen” sample) for comparison with the sonicated samples (referred to as the “sonicated” sample). Amplification employed cycles of sonication and incubation in a programmable sonicator (Qsonica, model Q-700) at 37 °C. A total of 96 PMCA cycles were performed comprising 20 s of sonication (at an amplitude of 38%, wattage: 278–299) followed by 29 min 40 s of incubation for each cycle. Frozen and sonicated samples were digested with proteinase K, and analysed by Western blotting using the monoclonal antibody 3F4 as described previously [1]. Amplification efficiency was expressed as the increase in PrP^res^ signal in the sonicated compared to the frozen sample, as judged by densitometry of Western blot images acquired and analysed using the ChemiDoc™ XRS + System with Image Lab Software (Bio-Rad, Hemel Hempstead, UK).

### Statistics

In the statistical analysis of the clinical patient information statistical significance was determined between the different *PRNP* codon 129 genotypes using a one-way ANOVA test followed by the Tukey’s multiple comparisons test. Statistical analysis of all clinical data was performed using GraphPad Prism software 7.00. In the statistical analysis of RT-QuIC data, a two sided Mann–Whitney test was used to assess the statistical significance of differences between the median RT-QuIC lag periods of sCJD versus hGH-iCJD or hDM-iCJD cases. The Kruskal–Wallis test was used for the combined analysis of sCJD (VV2, MV2, MM1) and hGH-iCJD (VV2, MV2) subgroups. These non-parametric tests were employed on the basis that the shapes of the distributions of the lag periods were similar for various subgroups. Statistical analysis and graphing of the data was performed using GraphPad Prism 7.00.

## Results

### *PRNP* codon 129 genotype of the hGH-iCJD cases

**Fig. 1:**
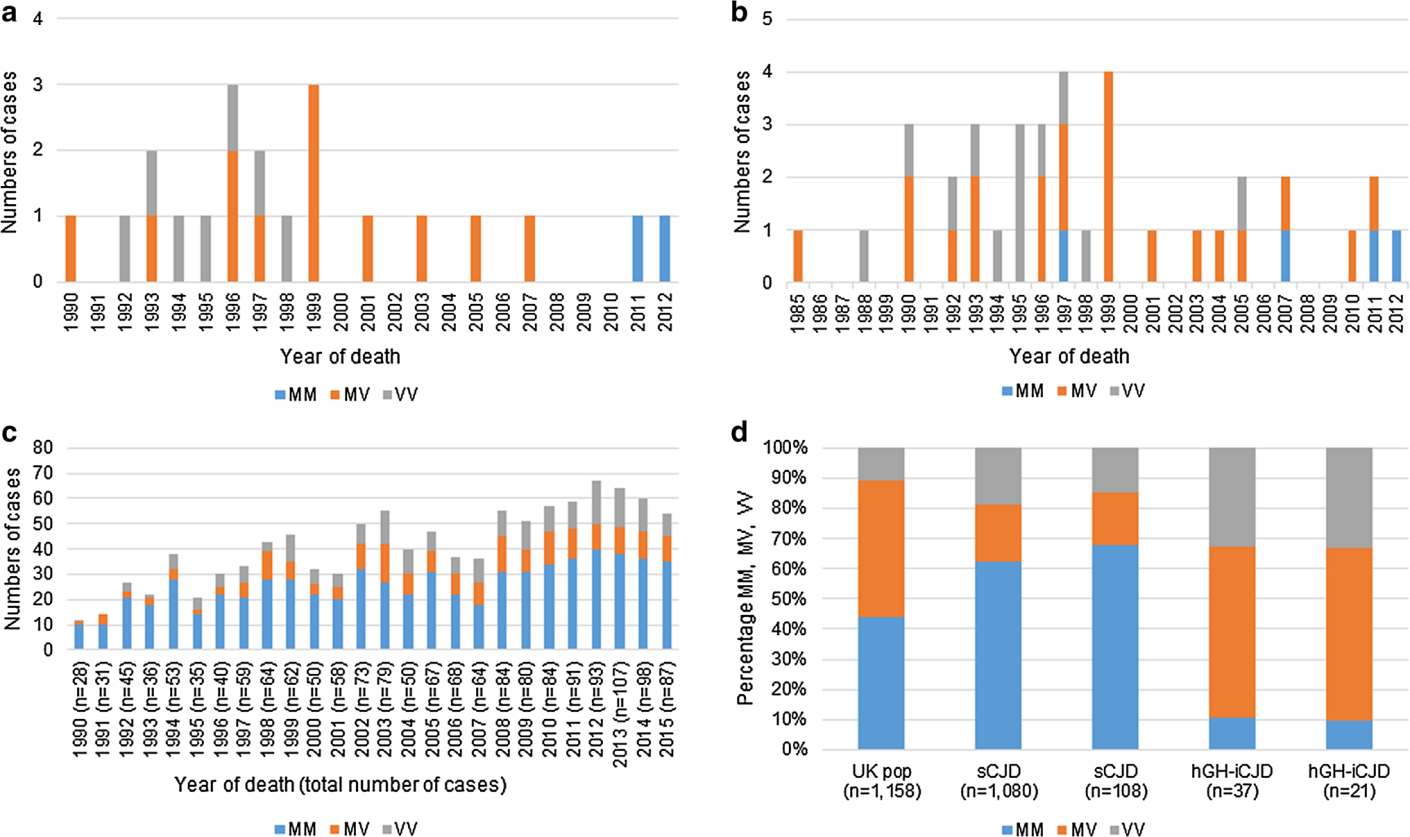
*PRNP* codon 129 genotype of hGH-iCJD and sCJD patient groups. **a** Shows the *PRNP* codon 129 distribution of hGH-iCJD cases by year of death for the 21 cases investigated in this study. **b** Shows the *PRNP* codon 129 distribution of hGH-iCJD cases by year of death for the 37 cases for which *PRNP* codon 129 data is available to us. **c** Shows the *PRNP* codon 129 distribution by year of death for the 1080 UK sCJD cases from 1990 to 2015. **d** Compares the *PRNP* codon 129 distribution of the hGH-iCJD (*n* = 21) and sCJD (*n* = 108) cases with frozen tissue analysed in this study, with the cases shown in **b** and **c**, of which they are a subset. The UK normal population *PRNP* codon 129 genotype distribution (UK pop), is shown for reference

There have been 78 cases of hGH-iCJD identified in the UK from 1985 to the present day. 43 of these had fixed and/or frozen tissue referred for diagnosis at the NCJDRSU. Of these 43 cases, 40 were autopsy cases and three were biopsy cases. 37 of these 43 cases had available codon 129 genotype data. 21 of the autopsy cases had frozen CNS tissues that were available for research use. The study identification numbers with clinical and pathological features of these 21 autopsy cases are shown in Online Resource Tables 1 and 2, respectively. When the hGH-iCJD patient *PRNP* codon 129 status is considered by year of death the following becomes apparent: first, the 21 cases of hGH-iCJD with frozen tissue analysed in this study died during the period from 1990 to 2012, and consequently these cases provide examples from the leading edge, the peak and the tail of the epidemic (Fig. 1a, b). Second, the UK hGH-iCJD group is dominated by patients of the MV and VV *PRNP* codon 129 genotypes; the few hGH-iCJD cases that occurred in individuals with an MM *PRNP* codon 129 genotype tended to occur later than those of the MV and VV *PRNP* codon 129 genotypes (Fig. 1a, b). In contrast, the MM *PRNP* codon 129 genotype is the most frequently occurring group within the UK sCJD group overall, and in every year during the hGH-iCJD epidemic (Fig. 1c). Finally, when considered as a whole, the distribution of codon 129 genotypes of the 21 available hGH-iCJD cases and the 108 sCJD cases selected for study here differ from each other and from the normal UK population, but they appear to be broadly representative subsets of the larger hGH-iCJD and sCJD cohorts (Fig. 1d). The three hDM-iCJD cases were all of the *PRNP* codon 129 MM genotype (Online Resource Table 2).

### hGH-iCJD patient information in relation to *PRNP* codon 129 genotype group

Age at death, duration of hGH treatment and estimated incubation periods were similar between the *PRNP* codon 129 MV and VV genotype hGH-iCJD groups, but the incubation periods in the two MM cases were longer. In contrast, the mean disease duration of illness of the MV group was longer than that of both the VV and MM groups (Online Resource Table 3).

### PrP^res^ types found in the hGH-iCJD cases

**Fig. 2:**
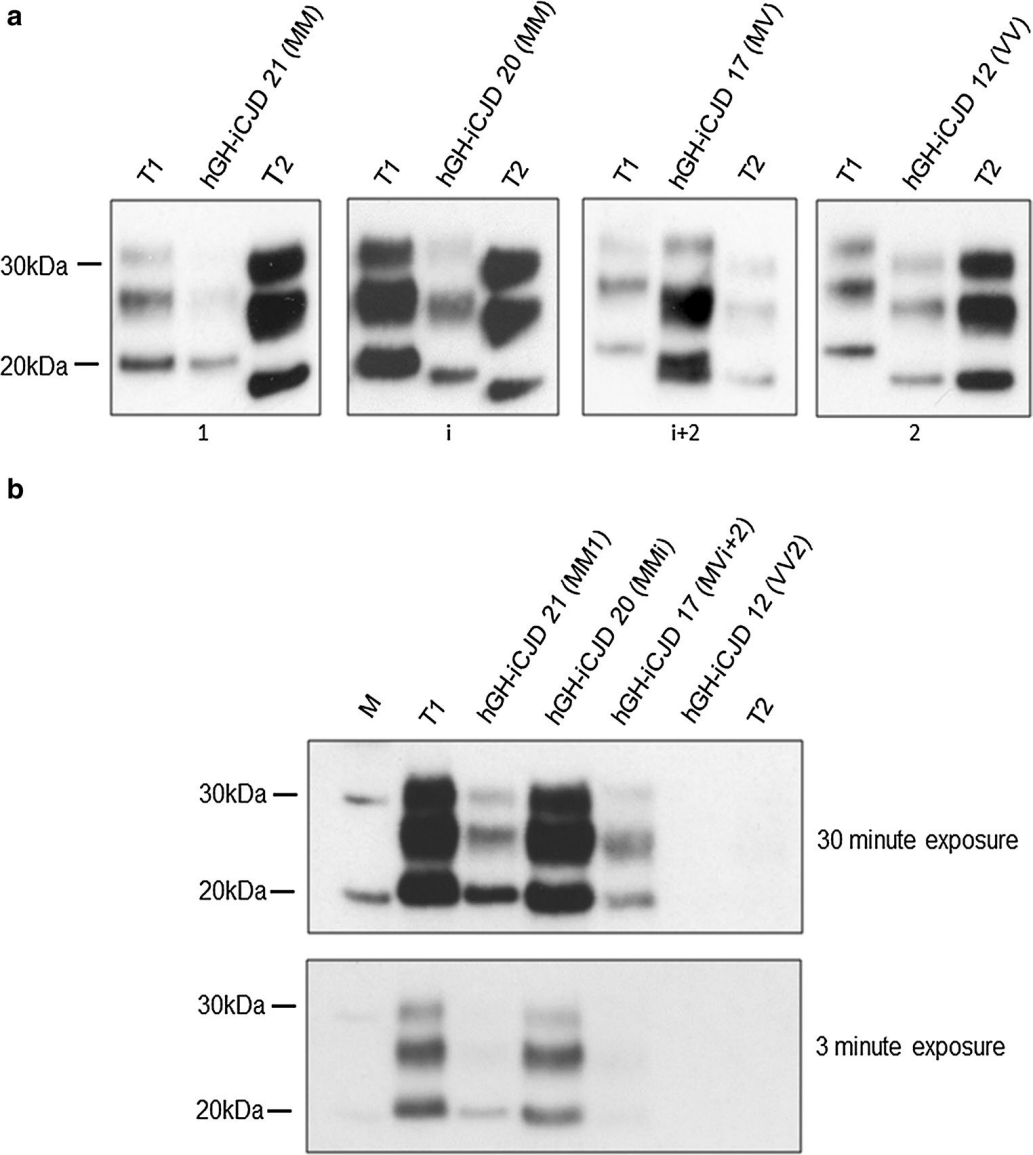
PrP^res^ type found in the iCJD cases examined. **a** Four PrP^res^ molecular types were detected by Western blot analysis of proteinase K treated brain extracts of hGH-iCJD patients; type 1, type i, type i + 2 and type 2. Representative blots of each PrP^res^ type are shown. Each sample (*middle lane*) is flanked by type 1 (T1) (*left lane*) and type 2 (T2) (*right lane*) reference standards from sCJD MM1 and VV2 subtype cases, respectively. Case number and codon 129 *PRNP* genotype are indicated above each blot, whilst PrP^res^ type is indicated below. The most informative exposure for the test and reference standard samples from a series of timed exposures is shown. Blots were probed using the monoclonal antibody 3F4. **b** Western blot analysis of proteinase K treated samples of PrP^res^ types detected in hGH-iCJD patients using the monoclonal antibody 12B2 which detects type 1 PrP^res^ and shown after a prolonged (30 min) and abbreviated (3 min) exposure time. Approximate molecular mass is shown in kDa. The immunoblots shown are representative of at least three technical repeats

**Fig. 3:**
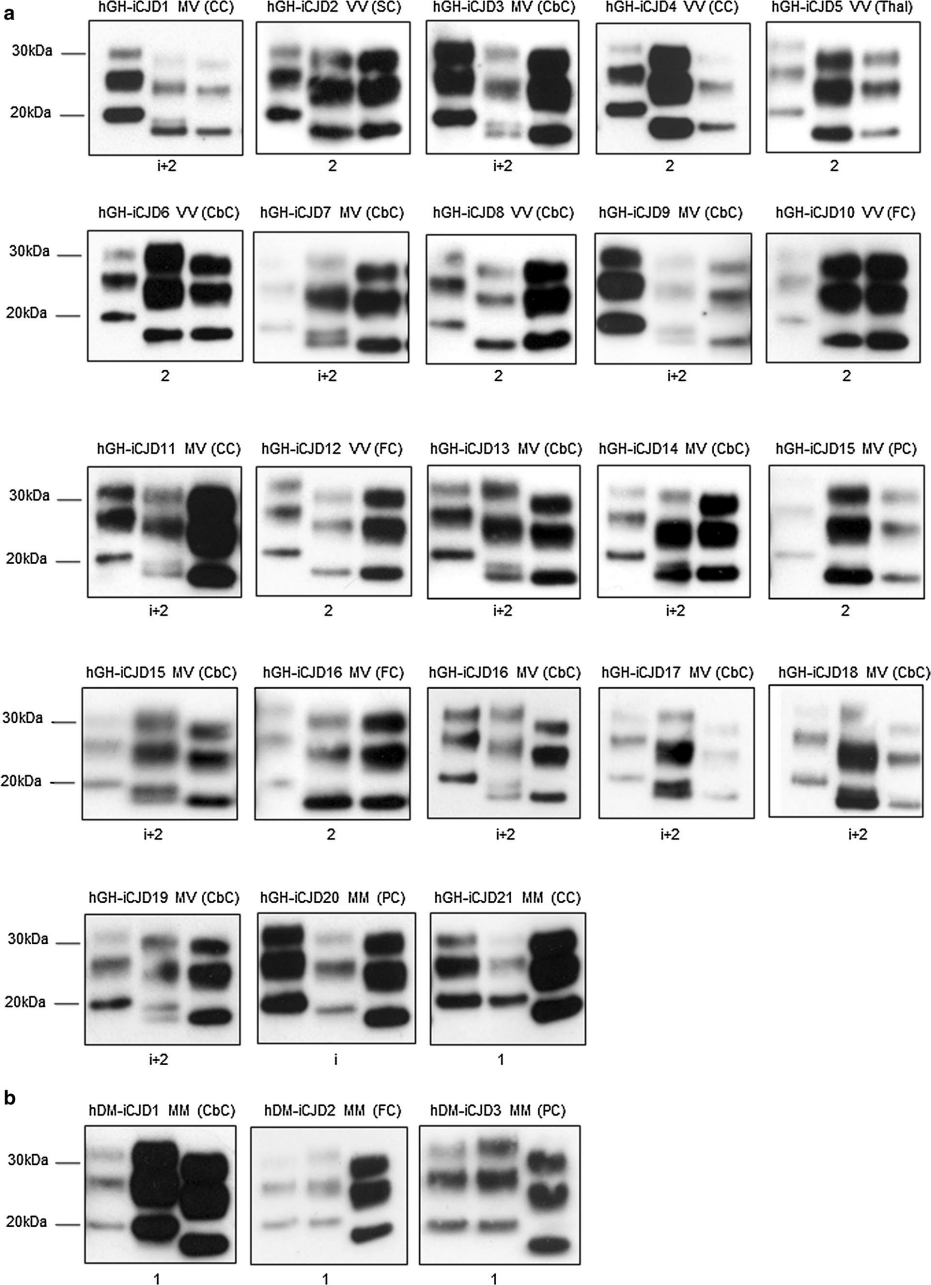
PrP^res^ type found in each individual iCJD case examined. PrP^res^ typing of (**a**) hGH-iCJD cases 1–21 and (**b**) hDM-iCJD cases 1–3. Each sample (*middle lane*) is flanked by a type 1 (*left lane*) and type 2 (*right lane*) reference standard from sCJD MM1 and VV2 subtype cases, respectively. Study identification number, *PRNP* codon 129 genotype and brain region are indicated above each blot and PrP^res^ type is indicated below. Approximate molecular mass is shown in kDa. The immunoblots shown are representative of at least three technical repeats

Western blot analysis of all available CNS tissue from the 21 cases of hGH-iCJD for protease resistant prion protein (PrP^res^) was performed. Four PrP^res^ types were identified in the hGH-iCJD cases, differing in the mobility and in the number of unglycosylated bands found. Representative examples of the PrP^res^ types 1, i, i + 2 and 2 in these cases are shown in Fig. 2a, example Western blots for each individual case are shown in Fig. 3, and the data compiled with the *PRNP* codon 129 genotype are shown in Online Resource Tables 1 and 2. The majority of cases were characterised by the presence of the 19 kDa type 2 PrP^res^ band (19/21 cases). However, in 12 out of these 19 cases the 20 kDa type i PrP^res^ band was also present. The relative abundance of the 19 kDa type 2 PrP^res^ band and the 20 kDa type i PrP^res^ band was variable, but the 19 kDa type 2 PrP^res^ band usually predominated (Fig. 3a). Type i was found to predominate overall in only one hGH-iCJD case (hGH-iCJD20) and the 21 kDa type 1 PrP^res^ was found in only one hGH-iCJD case (hGH-iCJD21). In contrast to the hGH-iCJD cases, all three cases of hDM-iCJD cases had type 1 PrP^res^ exclusively (Fig. 3b). Where it could be assessed, regional variation within the brain was largely confined to the presence or absence of the PrP^res^ type i band and the relative abundance of PrP^res^ types i and 2 bands (Online Resource Table 4). Comparison of the immunoreactivity of the 21 kDa type 1, 20 kDa type i, and 19 kDa type 2 bands using the 3F4 antibody (epitope 106-112) with the 12B2 antibody (epitope 89-93) showed that the 21 kDa type 1 PrP^res^ and the 20 kDa type i PrP^res^ fragments both retain the 12B2 epitope, whereas type 2 PrP^res^ fragments do not (Fig. 2a, b). Analysis of the PrP^res^ types found in the CNS of 108 sCJD cases showed type 1, type 2, type i + 2 and cases in which type 1 and type 2 were found in individual brains, either in the same or in different regions (Online Resource Table 5). These latter cases were designated type 1 + 2, irrespective of the relative abundance and location of the two types. The breakdown of these 108 sCJD cases according to the combination of CNS PrP^res^ type and *PRNP* codon 129 genotype is shown in Online Resource Table 6, while the histotypes and PrP^res^ types associated with the *PRNP* codon 129 MV sCJD cases are shown in Online Resource Table 7.

### PrP^res^ type and *PRNP* codon 129 genotype of the hGH-iCJD cases

**Fig. 4:**
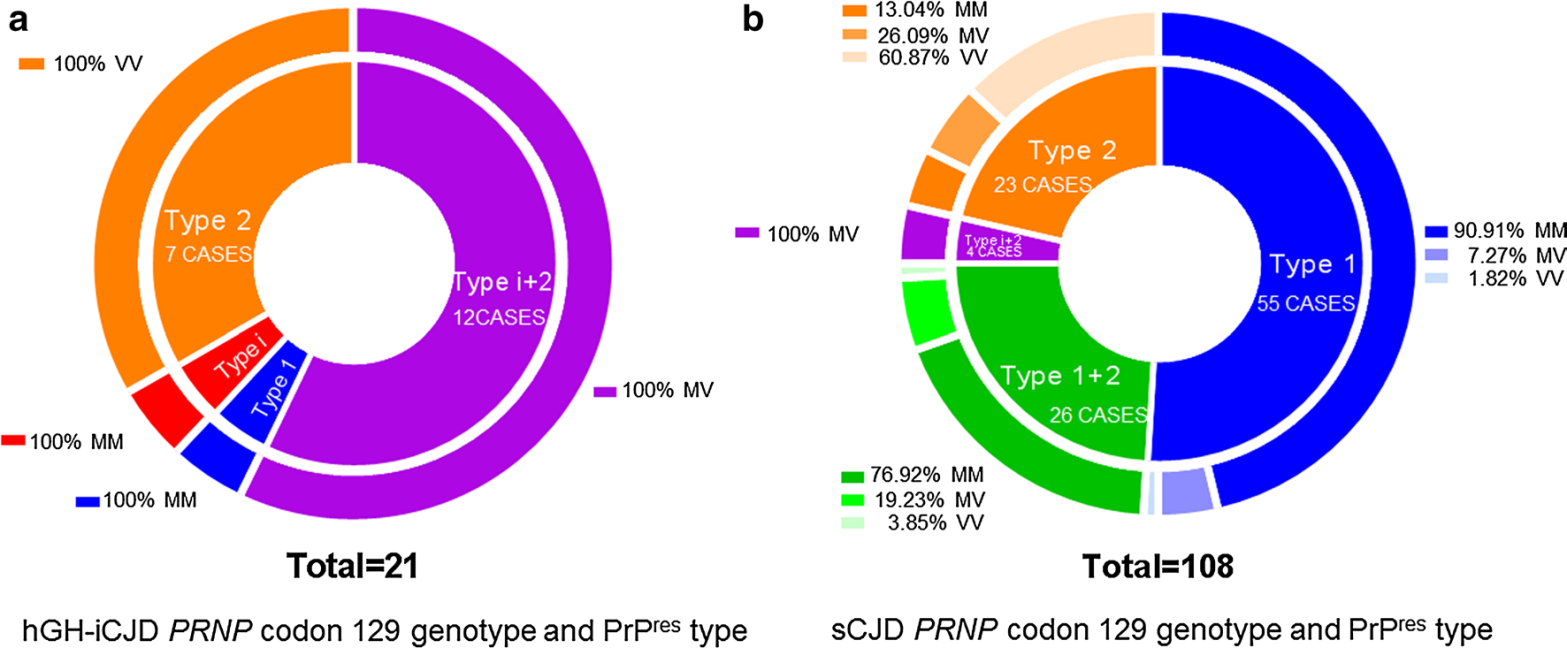
PrP^res^ type and *PRNP* codon 129 genotype of the hGH-iCJD and sCJD groups. **a** Shows the numbers of cases found within the hGH-iCJD group (*n* = 21) according to the combination of PrP^res^ type (*inner ring*) and *PRNP* codon 129 genotype (*outer ring*). **b** Shows the numbers of cases found within the sCJD group (*n* = 108) according to the combination of PrP^res^ type (*inner ring*) and *PRNP* codon 129 genotype (*outer ring*)

When the 21 cases of hGH-iCJD were grouped according to the combination of *PRNP* genotype and CNS PrP^res^ type (Online Resource Table 2; Fig. 4a) all *PRNP* codon 129 VV hGH-iCJD cases (7/7) were found to have type 2 PrP^res^ exclusively. All of the hGH-iCJD *PRNP* codon 129 MV genotype cases were found to have type i + 2 PrP^res^ (12/12). Of the two *PRNP* codon 129 MM genotype cases found within the hGH-iCJD cohort, one had type 1 PrP^res^ and the other had type i PrP^res^. In contrast, within the 108 sCJD cases, those with type 1 PrP^res^ and a *PRNP* codon 129 MM genotype predominated (50/108) and cases with type 1 + 2 PrP^res^ were relatively common (26/108), but no examples of type i PrP^res^ alone were found (Online Resource Table 6; Fig. 4b). The three hDM-iCJD cases were in individuals of the *PRNP* codon 129 MM genotype and each had PrP^res^ type 1 in the CNS (Fig. 3b).

### Neuropathology of hGH-iCJD

**Fig. 5:**
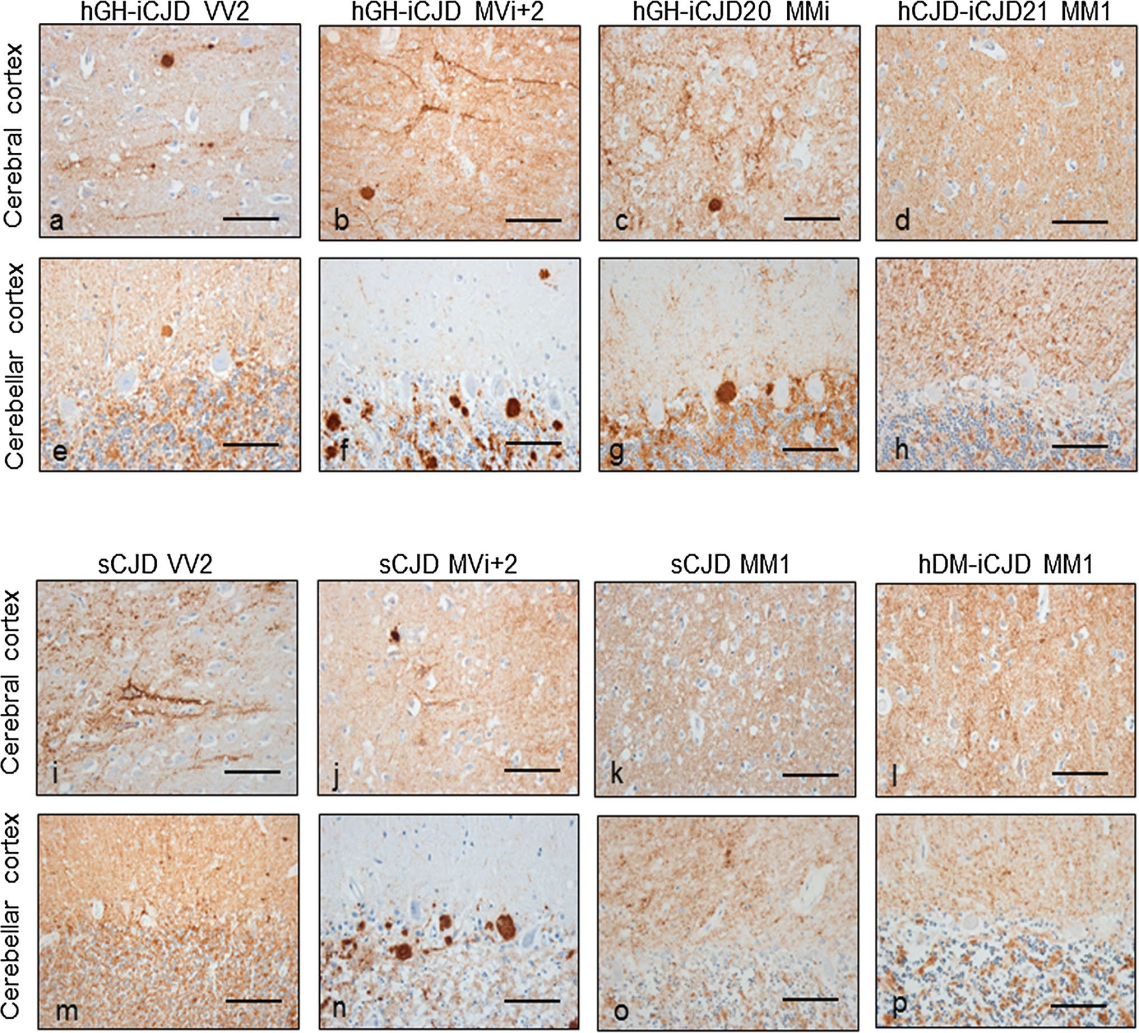
Characteristic neuropathology of the sCJD and iCJD cases. Prominent patterns of PrP pathology in the cerebral and cerebellar cortex in UK hGH-iCJD (**a**–**h**), sCJD (**i**–**k**, **m**–**o**) and hDM-iCJD patients (**l**, **p**). *PRNP* codon 129 VV hGH-iCJD patients showed a combination of synaptic, perineuronal and plaque-like deposits of PrP in the cerebral and cerebellar cortex (**a**, **e**). Intensely labelled kuru plaques and smaller plaque-like deposits were prominent features of the cerebral and cerebellar cortex in *PRNP* codon 129 MV hGH-iCJD patients (**b**, **f**). Contrasting patterns of PrP pathology were observed in the two *PRNP* codon 129 MM hGH-iCJD patients. Patient hGH-iCJD20 showed plaque-like deposits in the cerebral cortex (**c**) and numerous kuru plaques in the cerebellum (**g**) whereas hGH-iCJD21 showed only synaptic PrP deposits (**d**, **h**). The VV2 subtype in sCJD shows a perineuronal and synaptic pattern of PrP deposits in the cerebral and cerebellar cortex (**i**, **m**). The MV2 subtype of sCJD shows a synaptic and perineuronal deposition of PrP in the cerebral cortex (**j**) with kuru plaques and plaque-like deposits of PrP a dominant feature in the cerebellum (**n**). A widespread synaptic pattern of PrP accumulation is the characteristic feature of the MM1 subtype of sCJD (**k**, **o**). The two UK hDM-iCJD patients were also characterised by a predominantly synaptic pattern of PrP accumulation in the cerebral and cerebellar cortex (**l**, **p**). The anti-PrP antibody KG9 was used. *Scale bars* 50 µm

The neuropathological features were reviewed in the 21 hGH-iCJD patients (fixed tissue from case hGH-iCJD1 was not available for PrP immunohistochemistry). All cases showed a general widespread spongiform encephalopathy of a predominantly microvacuolar type accompanied by neuronal loss and gliosis, with amyloid plaques in the cerebellum in 13/21 cases. The patterns of accumulation of the disease-associated prion protein on immunohistochemistry were recorded in the 20 cases with available material. A summary of the neuropathological features of each hGH-iCJD case is added to the *PRNP* codon 129 genotype and PrP^res^ type in Online Resource Table 2 and example micrographs are shown in Fig. 5 (the neuropathological features in this cohort will be described more fully in a separate manuscript). Overall, the patterns of pathology in the hGH-iCJD patients showed close similarities with sCJD patients of the corresponding *PRNP* codon 129 genotype.

Immunohistochemistry on tissue sections from *PRNP* codon 129 VV hGH-iCJD patients showed a combination of synaptic, perineuronal and plaque-like deposits of PrP in the cerebral cortex often in a distinctive linear distribution in the deeper cortical layers, a pattern of PrP pathology very similar to that in the VV2 subtype of sporadic CJD (Fig. 5a, i). The cerebellum in these cases showed a coarser deposition of PrP with occasional plaque-like deposits (Fig. 5e, m). In contrast, all *PRNP* codon 129 MV hGH-iCJD patients were characterised by a pathology dominated by the accumulation of large and numerous kuru-type amyloid plaques in the cerebellar cortex and frequently in the cerebral cortex and basal ganglia, consistent with that observed in the sCJD MV2 subtype (Fig. 5b, f, j, n). The two hGH-iCJD *PRNP* codon 129 MM cases available for analysis showed strikingly different patterns of neuropathology. In patient hGH-CJD20, the spongiform change comprised a mixture of both microvacuolar and confluent types, with PrP staining showing a combination of synaptic, perineuronal and perivacuolar PrP deposits (Fig. 5c). In addition, plaque-like deposits and kuru plaques were a prominent feature in the cerebral and cerebellar cortex from this case (Fig. 5g). In contrast, hGH-CJD21 showed spongiform change of predominantly the microvacuolar type with a widespread synaptic/granular pattern of PrP deposition, a pattern more consistent with that observed in the sCJD MM1 subtype (Fig. 5d, h, k, o). In the two hDM-iCJD cases in which fixed tissue was available for immunohistochemical analysis, both showed a synaptic deposition of PrP throughout the brain. Kuru-type amyloid plaques or plaque-like PrP^res^ deposits were not observed in either case (Fig. 5l, p).

### Comparison of the amyloid seeding potential of hGH-iCJD and sCJD

The amyloid seeding activity of hGH-iCJD and sCJD cases was compared using the real-time quaking induced conversion assay (RT-QuIC). hGH-iCJD cases were selected on the basis of the availability of frozen cerebral cortex samples (*n* = 20). sCJD cases (*n* = 28) were selected on the basis of PrP^res^ type and *PRNP* codon 129 genotype to match, as far as possible, those found in the hGH-iCJD cohort. RT-QuIC assays were seeded with CJD cerebral cortex homogenate diluted by a factor of 2 × 10^−5^ (each 100 μl RT-QuIC reaction was seeded with the equivalent of 2 × 10^−7^ g brain) and the seeding activity for each patient was assumed to be inversely proportional to the mean lag period (Table 1). The hGH-iCJD and sCJD seed amounts were determined empirically to be within the range for quantifying seeding activity by the lag time method. The hGH-iCJD group as a whole (and their subgroups) have a lower median seeding activity than the sCJD group (and their matched subgroups). The difference is significant for the hGH-iCJD group as a whole and for the VV and MV subgroups. The two *PRNP* codon 129 MM hGH-iCJD cases had noticeably different lag times in this assay (hGH-iCJD20 at 43.5 h and hGH-iCJD21 at 27.5 h). Three hDM-iCJD cases were also analysed by RT-QuIC and their median lag period (34.2 h) was also greater than the sCJD MM1 subgroup, but this difference was not statistically significant. Although these experiments were conducted with recombinant hamster PrP, and therefore with a substrate containing methionine at the position equivalent to human codon 129, our previous experience with RT-QuIC indicates that it is less sensitive than PMCA to the biologically relevant effects of seed and substrate sequence matching or mismatching [26].

**Table 1:** Lag period analysis of RT-QuIC reactions seeded with cerebral cortex from cases of sCJD and iCJD

	sCJD	hGH-iCJD		sCJD	hGH-iCJD	sCJD	hGH-iCJD	sCJD		hDM-iCJD	hGH-iCJD	hGH-iCJD
Patient subgroup^a^	All subtypes			VV2	VV2	MV2^b^	MV2 + i	MM1		MM1	MM1	MMi
Median lag period^c^ (h)	14.7	22.0		14.9	27.4	12.3	17.9	15.0		34.2	27.5	43.5
Number of patients	28	20		11	6	10	12	7		3	1	1
IQR^d^	10.6–17.1	16.6–27.5		11.2–19.0	17.3–38.5	9.2–16.5	16.3–24.5	10.7–20.5		19.3–62.0	–	–
Mann–Whitney test^e^	*P* ≤ 0.0001			*P* = 0.027		*P* = 0.007		*P* = 0.067			–	–
Kruskal–Wallis test^f^	–	–		*P* = 0.005						–	–	–

^a^The patients were separated into subgroups according to *PRNP* codon-129 genotype and PrP^res^ type

^b^Cases in the sCJD MV2 group were chosen to be representative of all sCJD cases with an MV *PRNP* codon-129 genotype that have type 2 PrP^res^. Therefore, this group includes three type i + 2 cases, four type 2 cases and three type 1 + 2 cases

^c^The RT-QuIC period for each patient was calculated as the mean of at least four replicate analyses of one sample of cerebral cortex

^d^IQR = Interquartile range

^e^The Mann–Whitney test was used to assess the statistical significance of the difference in the median lag periods in pairwise comparisons of patient subgroups on the basis that the shapes of the distributions of lag periods were similar for the patient subgroups

^f^The Kruskal–Wallis test was used for the combined analysis of sCJD (VV2, MV2, MM1) and hGH-iCJD (VV2, MV2) subgroups

### Comparison of the in vitro amplification of hGH-iCJD and sCJD

**Fig. 6:**
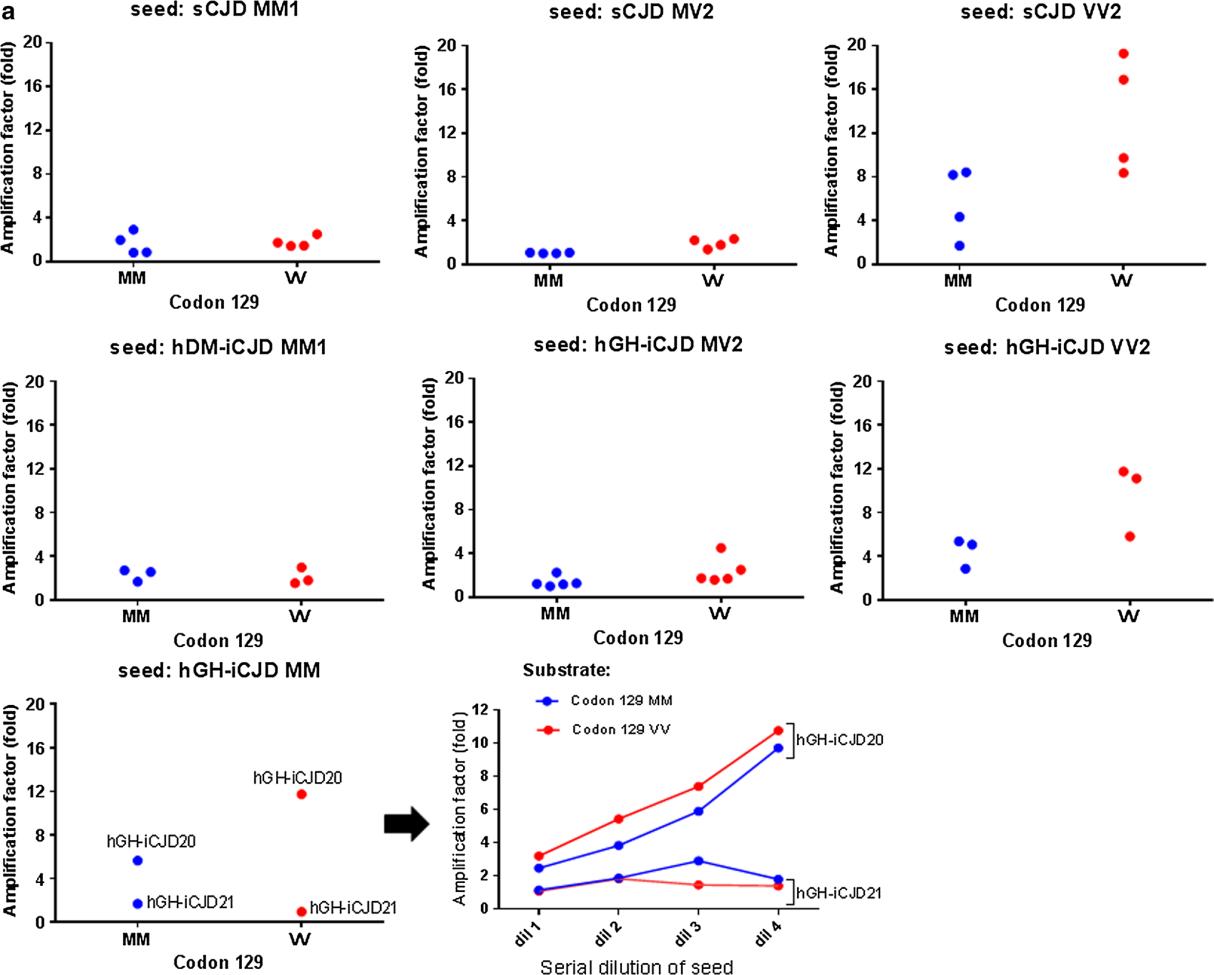

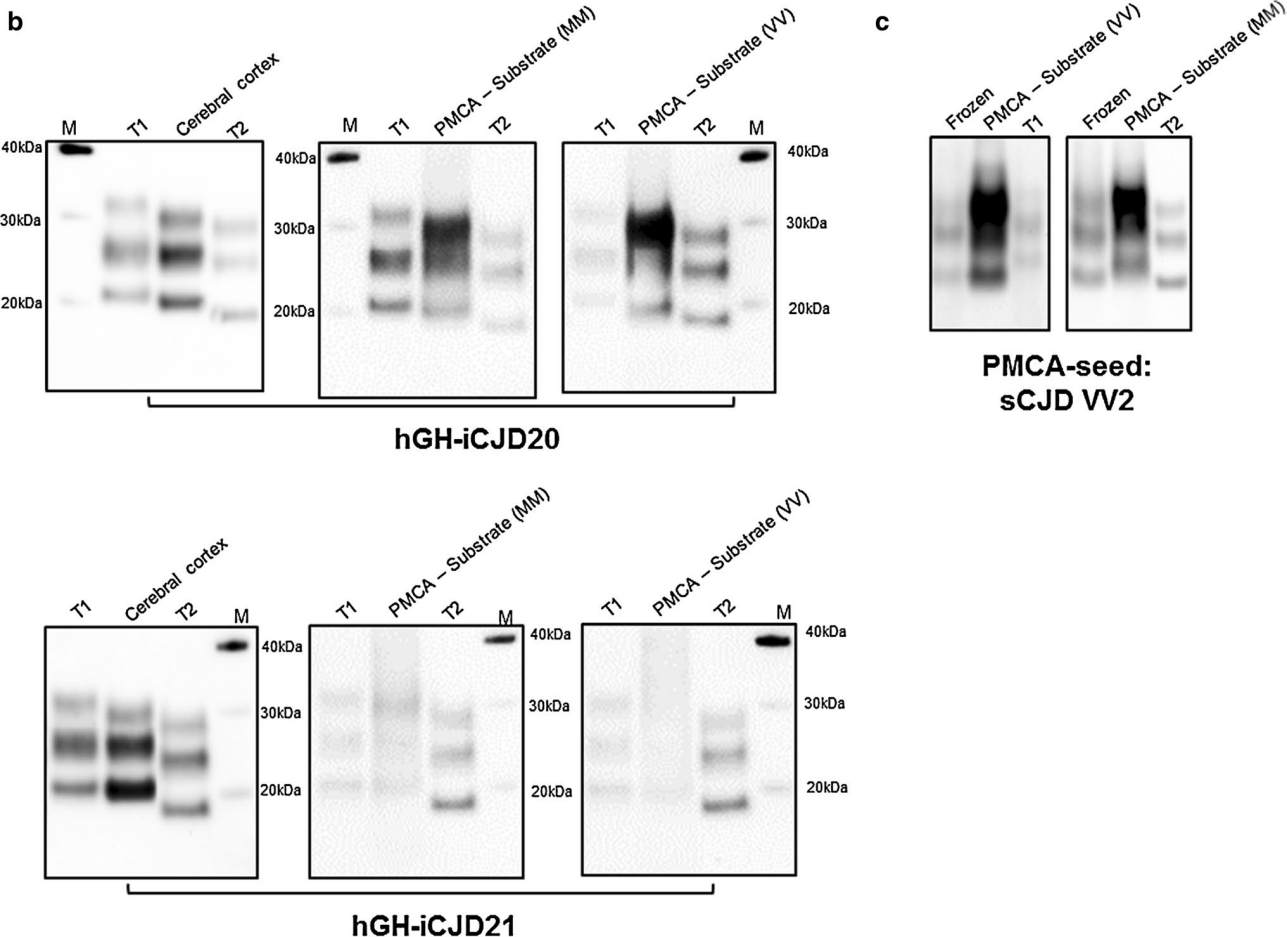
PrP^res^ analysis of PMCA reactions seeded with cerebral cortex from case of sCJD and iCJD. **a** hGH-iCJD, hDM-iCJD and sCJD brain homogenate (seeds) were diluted in humanised transgenic mouse brain homogenate (substrate) of the *PRNP* codon 129 MM (*red dots*) or VV (*blue dots*). Amplification factor is shown as the relative increase (fold) in PrP^res^ signal after a single round of PMCA as determined by densitometry of the Western blotting signal. PMCA reactions were normalized by seed PrP^res^ amount. PMCA of serial dilution of the hGH-iCJD MM seeds were also performed (hGH-iCJD20: 1/50, 1/100, 1/200, 1/400 and hGH-iCJD21: 1/4, 1/8, 1/16, 1/32). PMCA experiments seeded with hGH-iCJD, hDM-iCJD and sCJD samples were performed twice independently and repeated at least four times for the reactions seeded with the hGH-iCJD MM cases. **b** PrP^res^ type of MM hGH-iCJD cases 20 and 21 assessed before and after PMCA. PMCA reactions employed matched (MM) and mismatched (VV) *PRNP* transgenic mouse brain substrates. Each sample was run between a type 1 (*left lane*) and type 2 (*right lane*) reference standards from sCJD MM1 and VV2 subtype cases, respectively (T1 and T2). Study identification numbers of the hGH-iCJD MM cases are indicated. **c** PrP^res^ analysis of sCJD VV2 PMCA products detected by Western blot. sCJD VV2 brain homogenate was diluted and amplified in matched (MM) and mismatched (VV) codon 129 substrate by a single round of PMCA. Samples corresponding to unamplified (Frozen) and amplified (PMCA) aliquots are shown with reference standards as described above. A total of 25 biological replicates of hGH-iCJD, hDM-iCJD and sCJD were analysed

The seeding characteristics of the two divergent hGH-iCJD *PRNP* codon 129 MM cases (hGH-iCJD20 and hGH-iCJD21) were further examined using protein misfolding cyclic amplification (PMCA). Samples of cerebral cortex were used to seed matched (*PRNP* codon 129 MM, designated HuMM) and mismatched (*PRNP* codon 129 VV, designated HuVV) *PRNP* transgenic mouse brain substrates and the results compared with those of sCJD subtypes. PMCA reactions seeded with cerebral cortex samples from cases of sCJD of the MM1 subtype amplified poorly under the conditions used, whether in matched (MM) or mismatched (VV) humanised transgenic mouse brain substrate (Fig. 6a). This was also true of the three hDM-iCJD cases (hDM-iCJD1-3) (Fig. 6a). Seeding of PMCA reactions with cerebral cortex samples from cases of sCJD of the VV2 subtype amplified more efficiently under these same conditions, with the amplification levels higher in the matched (HuVV) than in the mismatched (HuMM) humanised transgenic mouse brain substrate (Fig. 6a). The two hGH-iCJD *PRNP* codon 129 MM cases gave divergent results: hGH-iCJD20 amplified well and more efficiently in the HuVV substrate, whereas hGH-iCJD21 failed to seed either substrate efficiently (Fig. 6a). This difference in amplification efficiency and substrate preference between hGH-iCJD20 and hGH-iCJD21 was independent of seeding dilution (Fig. 6a). In the case of hGH-iCJD20, the choice of *PRNP* codon 129 substrate (HuMM or HuVV) also had an effect on the PrP^res^ type of the PMCA product when analysed by Western blotting (Fig. 6b). The cerebral cortex sample from hGH-iCJD20 was PrP^res^ type i, but matching of the MMi seed with the HuMM substrate resulted in an upshift in mobility of the reaction product, whereas mismatching the MMi seed with the HuVV substrate resulted in an apparent downshift in mobility of the reaction product. hGH-iCJD21 amplified only poorly, and there was no evidence of any change in the type 1 PrP^res^ when the PMCA products were analysed by Western blotting (Fig. 6b). Addition examples of this reproducible, albeit subtle, mobility shift are shown (Online Resource Fig. 1). The possibility that PrP^res^ type i results from replication of the sCJD with type 2 PrP^res^ in an individual with a *PRNP* codon 129 MM genotype was then modelled using PMCA. When sCJD VV2 subtype brain homogenate was used to seed the HuMM substrate, an upward shift was seen, whereas amplification of the same seed in a *PRNP* codon 129 VV genotype resulted in conservation of the type 2 PrP^res^ (Fig. 6c). Further examples of this phenomenon using four different cases of sCJD VV2 subtype are shown (Online Resource Fig. 2).

### Comparison of the two hGH-iCJD *PRNP* codon 129 MM cases

**Fig. 7:**
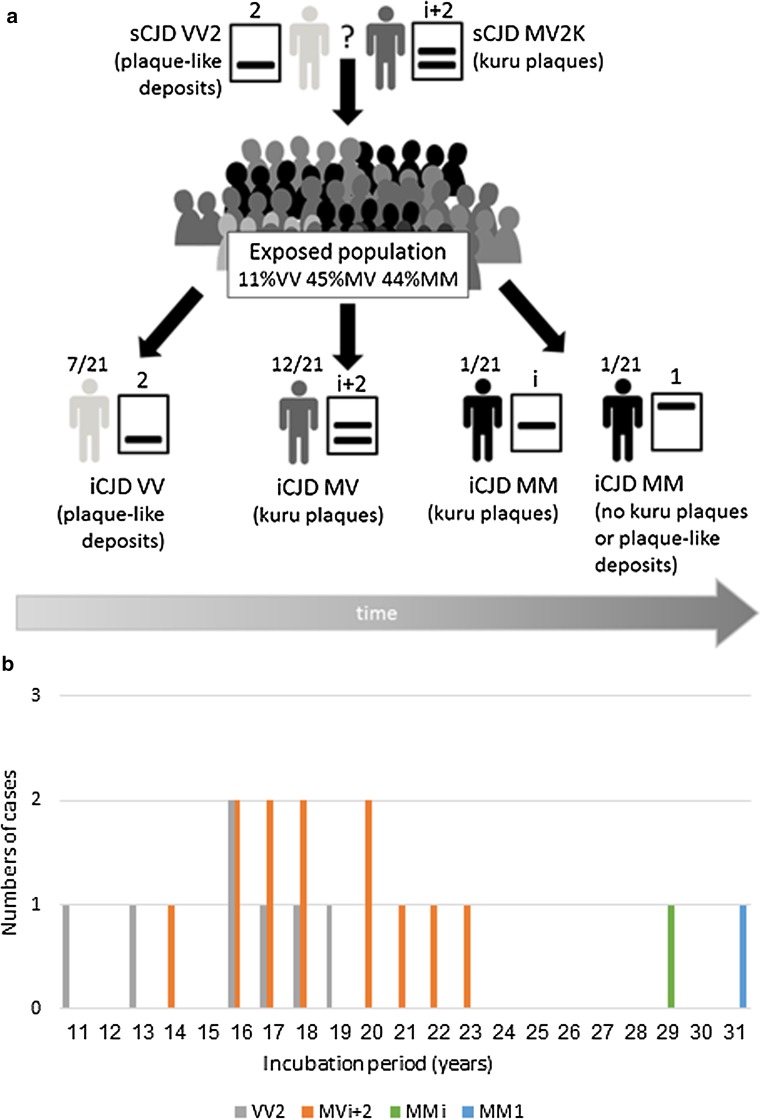
The UK hGH-iCJD epidemic and the study outcomes. **a** A summary of the molecular genetic finding of the hGH-iCJD cohort studied here showing correlations between *PRNP* codon 129 genotype, PrP^res^ type and pathology, the genetics of the exposed group and the suspected source of the hGH-iCJD infectivity. **b** Graphic representation of the numbers of hGH-iCJD subtypes (*PRNP* codon 129 genotype and PrP^res^ type) plotted according to increasing estimated incubation period

*PRNP* codon 129 MM is the rarest genotype within the UK hGH-iCJD cohort and cases with this genotype have tended to occur late during the epidemic. The two hGH-iCJD *PRNP* codon 129 MM cases available to this study were divergent in their neuropathology, PrP^res^ type and amplification potential in both the RT-QuIC and PMCA assays. In each of these respects, hGH-iCJD21 resembled the sCJD MM1 subtype (and the limited number of hDM-iCJD cases analysed), whereas hGH-iCJD20 more closely resembled the sCJD MV2 or VV2 subtypes, despite its *PRNP* codon 129 MM genotype. These data are summarised in Table 2 and Fig. 7.

**Table 2:** Comparison of two hGH-iCJD (MM) cases with differing pathological and molecular features

	hGH-iCJD21	hGH-iCJD20
*PRNP* codon 129 genotype	MM	MM
Neuropathology	Diffuse PrP positivity (resembling MM1 sCJD and hDM-iCJD)	PrP positive kuru plaques present (similar to MV2K sCJD)
RT-QuIC seeding activity	Shorter lag time (closer to MM1 sCJD)	Long lag time
PrP^res^	Type 1 (~21 kDa)	Type i (~20 kDa)
PMCA efficiency	Poor	Greater
PMCA substrate preference	None	VV > MM
PMCA product PrP^res^ mobility	Type 1	MM Substrate Results In Upshift VV Substrate Results In Downshift

## Discussion

This study provides a thorough molecular genetic description of the largest series of UK hGH-iCJD epidemic published to date, including as it does a large number of cases (21 compared to seven in a recent study [31]) in which the pathology, biochemistry and genetics can be compared. It also provides a description of the leading edge, peak and tail of the epidemic, rather than focussing largely on the tail of the epidemic [31]. In doing so, this study provides a unique opportunity to investigate the molecular epidemiology of prion transmission within a species, but across *PRNP* codon 129 polymorphic barriers.

The UK hGH-iCJD epidemic is widely thought to result from contamination of hGH batches with prions present in tissue (either pituitary or brain) collected from one, or more cases of “classical” Creutzfeldt-Jakob disease. On the balance of probabilities, this is more likely to be a form of sCJD (rather than a genetic form) since sCJD accounts for ~85% of human prion disease cases [12]. Although it cannot be known with certainty, the thesis proposed here is that the epidemiology of the epidemic is consistent with contamination with a single major prion strain.

Prion transmission (susceptibility, incubation period and phenotype) can be considered as an interaction between agent strain and host genotype, mediated in part by the related properties of abnormal prion protein conformation (for which PrP^res^ type is a surrogate marker) and the *PRNP* polymorphism that the encodes the residue at position 129 of the prion protein (either M or V). At present, five sCJD prion strains (designated M1, M2(c), M2(t), V1 and V2) have been distinguished by transmission studies in *PRNP* humanised transgenic mice and nonhuman primates and the V2 strain has been proposed to account for both MV2 and VV2 sCJD [3, 21, 24]. The surfeit of *PRNP* codon 129 MM hGH-iCJD cases in France, compared to the predominance of *PRNP* codon 129 VV hGH-iCJD cases in the UK as of 2003 was suggested to be attributable to different contamination events by different strains of agent [4]. Given that the two most frequently occurring subtypes of sCJD are the MM1 and VV2 subtype, the simplest hypothesis is that the French hGH-iCJD epidemic was caused primarily by contamination with MM1 sCJD (i.e. the M1 sCJD strain) and the UK hGH-iCJD epidemic caused primarily by VV2 or MV2 sCJD contamination (i.e. the V2 sCJD strain).

The data presented here and those of Rudge et al. [31] are consistent with that explanation, coupled with the *PRNP* genotype frequency of the at-risk hGH treated patient group, which if similar to UK control (e.g. blood donor) groups would be 44% MM, 45% MV and 11% VV [2]. Hence, the early surfeit of *PRNP* codon 129 VV UK hGH-iCJD cases could reflect the absence of a genotypic barrier and shorter incubation periods, albeit in a small subset of the hGH treated patient group. The appearance of more numerous cases in *PRNP* codon 129 MV individuals may reflect the larger numbers of *PRNP* codon 129 MV individuals in the hGH treated patient group. The appearance of a limited number of *PRNP* codon 129 MM hGH-iCJD cases, comparatively late in the epidemic, even although the hGH treated patient group would be expected to be ~40%, could be interpreted as evidence of a substantial *PRNP* codon 129 genotypic barrier to transmission of sCJD of the VV2 or MV2 subtypes, or a very prolonged incubation period. Although we cannot completely discount a mixture of contaminating strains including M1 in the form of sCJD MM1/MV1 subtype, the late appearance of these MM cases argues against a second minor M1 contaminating strain.

Our comprehensive data set coupled with the use of in vitro molecular conversion systems can be used to refine that model and provides a possible molecular mechanism for cross-genotype transmission. Within sCJD cases with *PRNP* codon 129 MV and PrP^res^ type 2, some cases have kuru plaques (the MV2K subtype), whereas others do not (the MV2C subtype). The MV2K sCJD subtype is characterised by PrP^res^ type i + 2 (a doublet of approximately 20 kDa and 19 kDa), whereas the MV2C subtype is reported to be characterised by the 19 kDa band only, and termed type 2 [17, 23, 24]. We note that all *PRNP* codon 129 MV UK hGH-iCJD cases had PrP^res^ type i + 2 (12/12) and kuru plaques. One of the two *PRNP* codon 129 MM hGH-iCJD cases described (hGH-iCJD20) also had kuru plaques (not normally found in *PRNP* codon 129 MM sCJD cases) and an intermediate ~20 kDa PrP^res^ type (type i), perhaps suggestive of a causal link to the MV2K subtype of sCJD. Moreover, the in vitro amplification characteristics of PrP^res^ from this case when used to seed the cell free prion protein conversion assay PMCA were not consistent with its *PRNP* codon 129 MM genotype, instead showing a preference for a substrate containing PrP^C^ with valine rather than methionine at position 129. This situation is highly reminiscent of the molecular trace-back phenomenon proposed by Kitamoto and coworkers to explain the phenotypic diversity of the MV2 sCJD cases, and the transmission properties and origin of dura mater-associated CJD in Japan [15, 16, 18]. Furthermore, Kobayashi et al. [19, 20] have proposed that kuru plaques in *PRNP* codon 129 MM apparent sCJD cases can be used as an indication of an iatrogenic rather than idiopathic/sporadic aetiology. The characterisation of our case hGH-iCJD20 here extends the trace-back phenomenon of Kitamoto and coworkers from dura mater-associated transmissions in Japan to UK growth hormone associated transmissions. One of our *PRNP* codon 129 MM hGH-iCJD cases has some similarities to one case recently described in a group of three hGH-iCJD cases in the USA [6]. The association of a *PRNP* codon 129 MM genotype with kuru plaques in three national independent iCJD cohorts adds weight to the trace-back hypothesis. Moreover, our data using PMCA also confirms that evidence of the trace-back phenomenon in hGH-iCJD can be acquired from cell free systems, in addition to animal transmission studies, as predicted for hDM-iCJD by Kobayashi et al. [20] and subsequently demonstrated by Takeuchi et al. [34].

The molecular mechanism of the trace-back phenomenon is not fully understood, but the data of Kitamoto and coworkers points to a key role for the intermediate PrP^res^ type, termed type i. In this light, it is also tempting to suggest that our data points to the sCJD MV2K subtype as the source of the UK hGH-iCJD epidemic. It is plausible that the combination of the 20 kDa type i and 19 kDa type 2 PrP^res^ conformational variants found in sCJD MV2K might engender enhanced genotypic host range according to the principles of the conformational selection model proposed by Collinge and Clarke [7]. In this scheme, the MV2K subtype would transmit efficiently to MV individuals resulting in hGH-iCJD with a recognisable MV2K phenotype involving kuru plaques. The presence of type 2 PrP^res^ in the type i + 2 isoform would also facilitate efficient transmission to *PRNP* codon 129 VV individuals and result in the recognisable VV2 phenotype lacking kuru plaques. Finally, the coexistence of type i with type 2 PrP^res^ within the type 2d or 2 doublet isoform might render MV2K more compatible with transmission to *PRNP* codon 129 MM individuals, but only with extended incubation periods and producing a phenotype with PrP^res^ type i and kuru plaques or, if molecular adaptation is complete, type 1 PrP^res^ and an MM1 phenotype.

Alternatively, a sCJD VV2 source of infection in the hGH-iCJD cohort would involve a molecular strain adaptation-based model (rather than a molecular strain selection-based model) in which the *PRNP* codon 129 polymorphism of the host imposes a modified PrP^Sc^ conformation on the infectious agent. The in vitro PrP^res^ type switching we observed when sCJD VV2 was amplified in a mismatched substrate (MM) is entirely consistent with this second VV2 source adaptation-based model. These scenarios are summarised in Fig. 7a and the epidemic is plotted according to estimated incubation periods in Fig. 7b. The most relevant published humanised transgenic mouse model transmission data do not help in resolving the VV2/MV2 question, because the VV2 sCJD and MV2 sCJD cases used transmitted with indistinguishable characteristics in these mice. However, it is noteworthy that the most efficient transmission with the shortest incubation period was with the V2 strain (both VV2 and MV2 sCJD) in *PRNP* codon 129 VV mice [3]. Distinguishing between sCJD VV2 and MV2K as the source of UK hGH-iCJD may be more than a formal difficulty given our recent finding that the proportion of PrP^res^ derived from the two *PRNP* M and V alleles can vary dramatically in sCJD, such that some heterozygous sCJD cases might in effect contain only PrP^res^ derived from the 129 V or the 129 M alleles [22]. Irrespective of mechanisms, the chronological sequence of cases (as judged by date of death and by estimated incubation period) involves a gradual change from predominantly *PRNP* codon 129 VV cases first, then MV cases during the peak and finally MM cases later and a corresponding change from predominantly PrP^res^ type 2, to more complex type i + 2 and then the two cases with PrP^res^ type i and type 1.

### Implications for person-to-person transmission

The RT-QuIC and PMCA comparisons performed here did not support the hypothesis that human-to-human transmission of CJD results in acquired replicative efficiency [36]. Indeed, the RT-QuIC assay showed reduced rather than enhanced seeding activity in hGH-iCJD compared to the matched subtypes of sCJD suggesting that hGH-iCJD is not markedly more of a public health concern than its presumed source, sCJD. However, CJD surveillance systems in countries where contaminated batches of growth hormone were used should remain vigilant for further cases of hGH-iCJD. Some of these cases may be identifiable by the presence of kuru plaques and the presence of PrP^res^ type i. However, others cases may appear closely similar to the most common form of sCJD (MM1), and the only indication of an iatrogenic aetiology may be in their medical history. The overriding message from the UK experience with hGH-iCJD and perhaps human prion diseases, more generally, is that poorly assessed risks can continue to have ramifications for patients years to decades after the risk source is itself recognised and removed. This point is underscored by a further case of hGH-iCJD being identified during the writing of this report in 2016, some 30 years after treatment with pituitary-derived hGH was abandoned in the UK.

## Electronic supplementary material

Below is the link to the electronic supplementary material.

Supplementary material 1 (DOCX 540 kb)
