# Supplementary material for: UK Iatrogenic Creutzfeldt–Jakob disease: investigating human prion transmission across genotypic barriers using human tissue-based and molecular approaches
Source: Acta Neuropathol. 2016 Nov 3;133(4):579–95. doi: 10.1007/s00401-016-1638-x (PMC5348565; doi:10.1007/s00401-016-1638-x)
Supplement: Supplementary file 1 — Supplementary material 1 (DOCX 540 kb) [file 401_2016_1638_MOESM1_ESM.docx]

**Acta Neuropathologica Online Resource**

**UK Iatrogenic Creutzfeldt-Jakob disease: Investigating human prion transmission across genotypic barriers using human tissue-based and molecular approaches**

Diane L. Ritchie, Marcelo A. Barria, Alexander H. Peden, Helen M. Yull, James Kirkpatrick, Peter Adlard, James W. Ironside, Mark W. Head

Author for correspondence: Dr Mark W. Head, National CJD Research & Surveillance Unit, Centre for Clinical Brain Sciences, Deanery of Clinical Sciences, The University of Edinburgh. E-mail: m.w.head@ed.ac.uk

**Figures**

**Fig. 1**

**
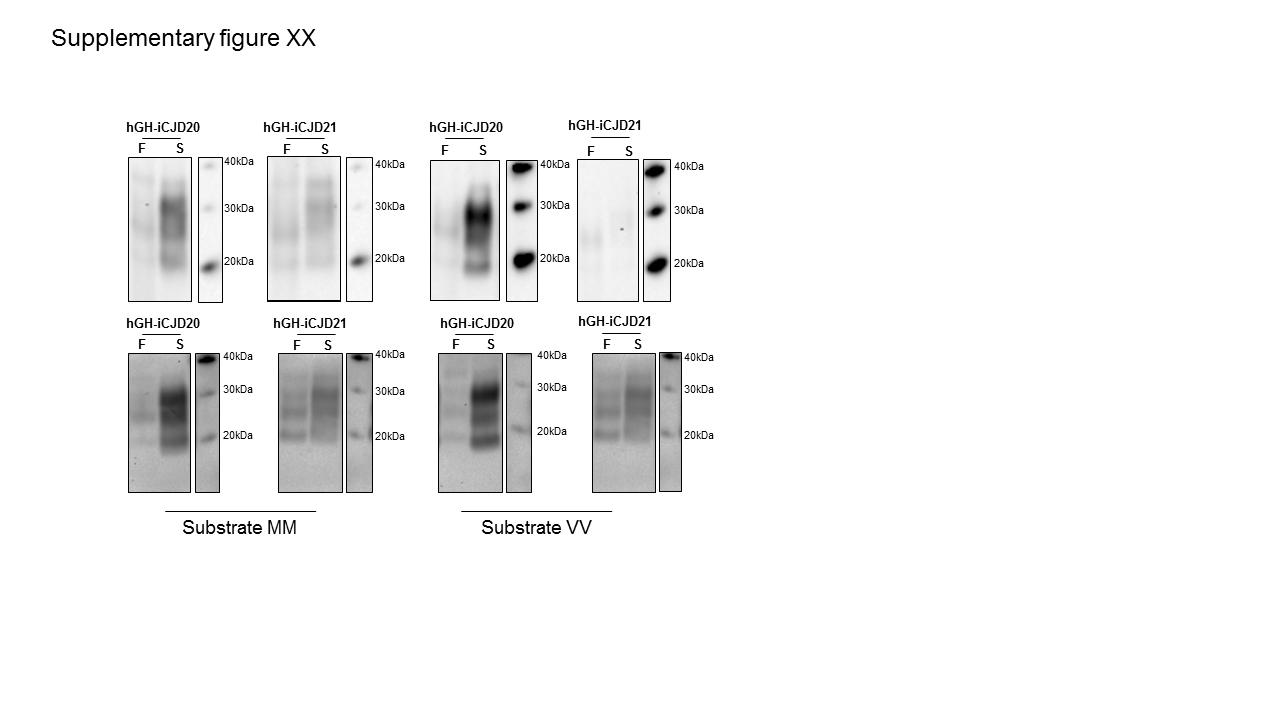
**

**Figure 1: PrP^res^ analysis of PMCA reactions seeded with hGH-iCJD MM cases.** Amplification of iatrogenic CJD human growth hormone (hGH-iCJD) MM1 and MMi cases. Brain homogenate (cerebral cortex) from hGH-iCJD20 and hGH-iCJD21 cases were incubated with humanised transgenic mouse brain homogenate (substrate) of the *PRNP* codon 129 MM and VV and amplified by PMCA. Reactions were normalized by seed PrP^res^ input diluted 1/100 and 1/8 for the hGH-iCJD20 and hGH-iCJD21 respectively. Independent experiments were performed and combined for presentation in the figure. The upper and lower section are from two independent experiments. The reactions were evaluated by Western blotting. Unamplified samples are designated “Frozen” sample (F) and amplified aliquots as “Sonicated” (S). The molecular mass of electrophoretic markers is given in kilodaltons (kDa).

**Fig. 2**

**
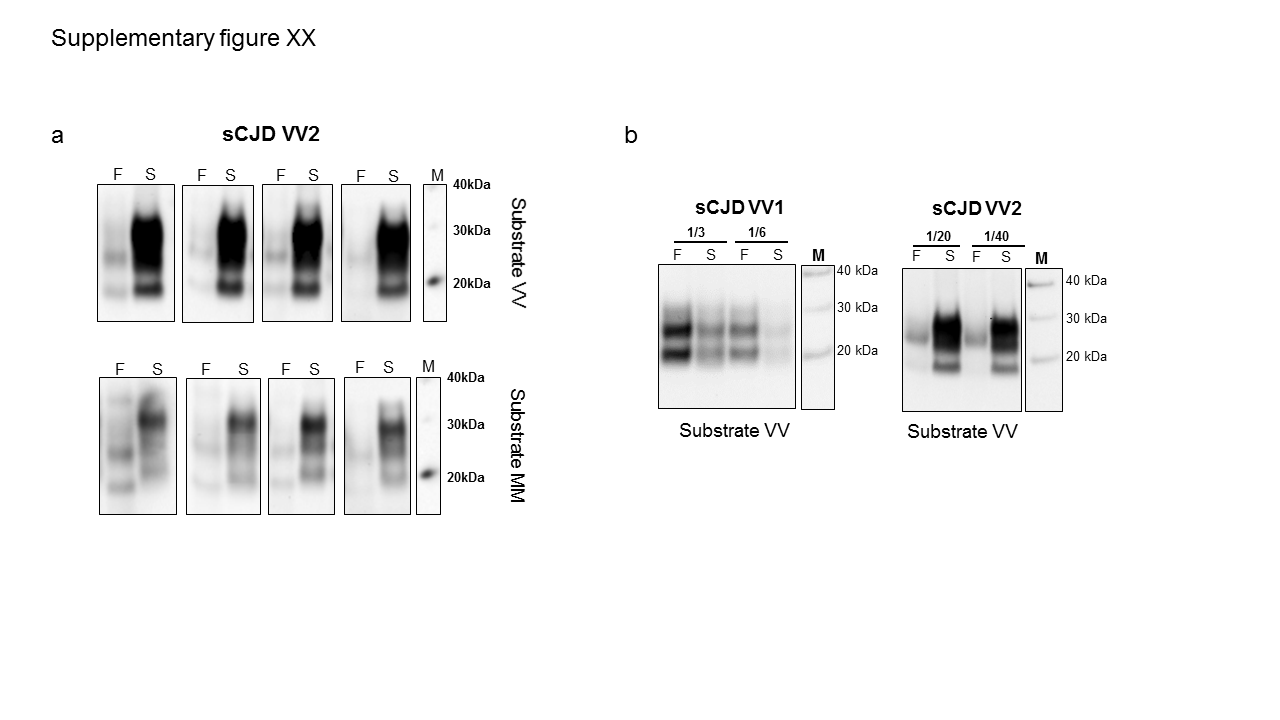
**

**Figure 2: PrP^res^ analysis of PMCA reactions seeded with sCJD VV2 and VV1 subtypes.** PMCA reactions seeded with cerebral cortex from cases of sCJD VV2 and VV1. (a) Brain homogenate from four sCJD VV2 cases were diluted in humanised transgenic mouse brain homogenate (substrate) of the *PRNP* codon 129 MM (top panel) and VV (lower panel) and subjected to PMCA. Reactions were normalised by seed PrP^res^ amount prior to the amplification process. (b) PMCA experiments seeded with sCJD VV1 and sCJD VV2 samples were also performed. Serial dilution of the sCJD VV1 and sCJD VV2 seeds were achieved using humanised transgenic mouse brain substrate codon 129 VV (sCJD VV1: 1/3, 1/6 and sCJD VV2: 1/20, 1/40). The reactions were evaluated by Western blotting. Unamplified samples are designated “Frozen” sample (F) and amplified aliquots as “Sonicated” (S). Molecular mass of electrophoretic markers is given in kilodaltons (kDa).

**Tables**

**Table 1: Basic patient information for the iCJD cases examined.**

| **Study ID** | **M/F** | **Age at death (years)** | **Age at onset in years**  **(year of onset)** | **Disease duration (months)** | **Duration of treatment in years or**  ***year of graft** | **Incubation period (years^a^)** | ***PRNP* codon 129** | **PrP^res^ type** |
| --- | --- | --- | --- | --- | --- | --- | --- | --- |
| hGH-iCJD1 | M | 34 | 33 (1989) | 8 | 4 | 17 | MV | i+2 |
| hGH-iCJD2 | M | 31 | 30 (1991) | 6 | 10 | 16 | VV | 2 |
| hGH-iCJD3 | M | 27 | 26 (1992) | 17 | 6 | 14 | MV | i+2 |
| hGH-iCJD4 | M | 30 | 29 (1992) | 9 | 4 | 13 | VV | 2 |
| hGH-iCJD5 | M | 25 | 25 (1993) | 5 | 6 | 11 | VV | 2 |
| hGH-iCJD6 | F | 33 | 32 (1994) | 6 | 4 | 18 | VV | 2 |
| hGH-iCJD7 | F | 31 | 30 (1995) | 12 | 4 | 16 | MV | i+2 |
| hGH-iCJD8 | M | 29 | 28 (1996) | 6 | 8 | 17 | VV | 2 |
| hGH-iCJD9 | M | 33 | 32 (1995) | 14 | 10 | 18 | MV | i+2 |
| hGH-iCJD10 | M | 36 | 36 (1996) | 8 | 4 | 19 | VV | 2 |
| hGH-iCJD11 | M | 37 | 35 (1996) | 18 | 4 | 18 | MV | i+2 |
| hGH-iCJD12 | M | 27 | 27 (1998) | 5 | 7 | 16 | VV | 2 |
| hGH-iCJD13 | M | 34 | 32 (1997) | 16 | 5 | 17 | MV | i+2 |
| hGH-iCJD14 | M | 29 | 28 (1997) | 15 | 9 | 16 | MV | i+2 |
| hGH-iCJD15 | M | 37 | 37 (1999) | 9 | 6 | 20 | MV | i+2 |
| hGH-iCJD16 | M | 30 | 28 (2000) | 16 | 3 | 20 | MV | i+2 |
| hGH-iCJD17 | F | 41 | 38 (2001) | 22 | 10 | 23 | MV | i+2 |
| hGH-iCJD18 | F | 34 | 32 (2003) | 23 | 8 | 22 | MV | i+2 |
| hGH-iCJD19 | F | 29 | 27 (2004) | 32 | 7 | 21 | MV | i+2 |
| hGH-iCJD20 | M | 46 | 46 (2011) | 5 | 3 | 29 | MM | i |
| hGH-iCJD21 | M | 42 | 42 (2012) | 8 | 8 | 31 | MM | 1 |
| hDM-iCJD1 | F | 45 | 44 (1990) | 5 | *1983 | 8 | MM | 1 |
| hDM-iCJD2 | F | 27 | 24 (1994) | 33 | * 1986 | 9 | MM | 1 |
| hDM-iCJD3 | M | 34 | 33 (2002) | 5 | * 1987 | 15 | MM | 1 |

1. Estimated as the period of time between the midpoint of hGH treatment and the onset of clinical symptoms

**Table 2: Pathological features of the iCJD cases examined.**

| **Study ID** | **Genotype (*PRNP* codon 129)** | **PrP^res^ type** | **Prominent neuropathological features** | **Histotype** |
| --- | --- | --- | --- | --- |
|  |  |  |  |  |
| hGH-iCJD1 | MV | i+2 | Microvacuolation with some large confluent vacuoles; neuronal loss and gliosis most severe in the cerebellum with kuru plaques prominent in the cerebellar cortex | MV2K+2C |
| hGH-iCJD2 | VV | 2 | Microvacuolation most prominent in deeper cortical layers; PrP staining shows synaptic and perineuronal deposits with plaque-like deposits in the cerebral and cerebellar cortex | VV2 |
| hGH-iCJD3 | MV | i+2 | Microvacuolation with synaptic and perineuronal PrP staining in all cortical layers. Kuru plaques and plaque-like deposits prominent in the cerebral and cerebellar cortex | MV2K |
| hGH-iCJD4 | VV | 2 | Microvacuolation with some focal confluent vacuoles; PrP staining shows synaptic and perineuronal deposits with plaque-like deposits in the cerebral and cerebellar cortex | VV2 |
| hGH-iCJD5 | VV | 2 | Prominent microvacuolation with perineuronal PrP staining in addition to plaque-like deposits in the cerebral cortex | VV2 |
| hGH-iCJD6 | VV | 2 | Prominent microvacuolation with perineuronal PrP staining in addition to plaque-like deposits in the cerebral cortex | VV2 |
| hGH-iCJD7 | MV | i+2 | Microvacuolation with some large confluent vacuoles; PrP staining shows synaptic and perineuronal PrP staining with kuru plaques and plaque-like deposits prominent in the cerebral and cerebellar cortex | MV2K+2C |
| hGH-iCJD8 | VV | 2 | Microvacuolation most prominent in deeper cortical layers with perineuronal PrP staining and numerous plaque-like deposits in the cerebral cortex | VV2 |
| hGH-iCJD9 | MV | i+2 | Microvacuolation with synaptic and perineuronal PrP staining in all cortical layers. Kuru plaques and plaque-like deposits prominent in the cerebral and cerebellar cortex | MV2K |
| hGH-iCJD10 | VV | 2 | Prominent microvacuolation in deeper cortical layers with synaptic and perineuronal PrP staining in addition to plaque-like deposits | VV2 |
| hGH-iCJD11 | MV | i+2 | Prominent microvacuolation with some confluent vacuoles in deeper cortical layers with perineuronal PrP staining in addition to plaque-like deposits. Kuru plaques prominent in the cerebellar cortex | MV2K +2C |
| hGH-iCJD12 | VV | 2 | Microvacuolation with PrP staining showing synaptic and perineuronal deposits with plaque-like deposits in the cerebral and cerebellar cortex | VV2 |
| hGH-iCJD13 | MV | i+2 | Microvacuolation with synaptic and perineuronal PrP staining. Kuru plaques and plaque-like deposits prominent in the cerebral and cerebellar cortex | MV2K |
| hGH-iCJD14 | MV | i+2 | Prominent microvacuolation in deeper cortical layers with perineuronal and synpatic PrP staining in addition to plaque-like deposits. Kuru plaques present in the cerebellar cortex | MV2K |
| hGH-iCJD15 | MV | i+2 | Prominent microvacuolation in deeper cortical layers with perineuronal PrP staining in addition to plaque-like deposits. Kuru plaques present in the cerebral and cerebellar cortex | MV2K |
| hGH-iCJD16 | MV | i+2 | Prominent microvacuolation in deeper cortical layers with perineuronal PrP staining in addition to plaque-like deposits. Kuru plaques present in the cerebral and cerebellar cortex | MV2K |
| hGH-iCJD17 | MV | i+2 | Prominent microvacuolation with PrP staining showing synaptic and perineuronal deposits in addition to plaque-like deposits. Kuru plaques present in the cerebellar cortex. | MV2K |
| hGH-iCJD18 | MV | i+2 | Microvacuolation with synaptic and perineuronal PrP staining. Kuru plaques and plaque-like deposits prominent in the cerebral and cerebellar cortex | MV2K |
| hGH-iCJD19 | MV | i+2 | Status spongiosis with collapse of cerebral architecture. Synaptic PrP staining in addition to plaque-like deposits. Kuru plaques present in the cerebellar cortex | MV2K |
| hGH-iCJD20 | MM | i | Microvacuolation with some large confluent vacuoles; PrP staining shows synaptic and perineuronal deposits in addition to plaque-like deposits. Kuru plaques present in the cerebral and cerebellar cortex. | Atypical – resembles MV2K+2C |
| hGH-iCJD21 | MM | 1 | Microvacuolation with synaptic PrP staining in cerebral and cerebellar cortex. | MM1 |
| hDM-iCJD1 | MM | 1 | Microvacuolation most marked in the cerebral cortex with relatively little cerebellar pathology. | MM1 |
| hDM-iCJD2 | MM | 1 | Status spongiosis with collapse of cerebral architecture. Intense synaptic PrP staining in cerebral and cerebellar cortex | MM1 |
| hDM-iCJD3 | MM | 1 | Microvacuolation with synaptic PrP staining in cerebral and cerebellar cortex. | MM1 |

**Table 3: Patient information in relation to *PRNP* codon 129 genotype group.**

|  | hGH-iCJD cases | | |
| --- | --- | --- | --- |
|  | MM (n=2) | MV (n=12) | VV (n=7) |
| Age at death in years (mean ± SD) | 44 ± 2.82  (46y, 42y) | 33 ± 4.05 | 30.2 ± 3.67 |
| Disease duration in months (mean ± SD) | 6.6 ± 2.12  (5m, 8m) | 16.8 ± 6.55 | 6.43 ± 1.51 |
| Duration of treatment in years (mean ± SD) | 5.5 ± 3.54  (2.8y, 8.1y) | 6.3 ± 2.46 | 6.14 ± 2.34 |
| Incubation period in years (mean ± SD) | 30 ± 1.41  (29.4y, 31.7y) | 18.5 ± 2.71 | 15.7 ± 2.81 |

No statistically significant difference was found between the different *PRNP* codon 129 genotype groups and duration of treatment. However, statistically significant differences were observed between genotype groups, in terms of the age at death, disease duration and incubation period using a one-way ANOVA (*p*=0.0012, *p*=0.0011, *p*<0.0001, respectively). When comparing the MV and VV groups, no statistical differences were observed for incubation period and age of death; however, differences were observed when comparing the MV and VV groups with disease duration (*p*=0.0015). Comparison of the MM and MV groups showed statistically significant differences in terms of disease duration (*p*=0.0461), incubation period (*p*<0.0001) and age at death (*p*=0.0042). Comparisons of the MM and VV genotypes observed differences with incubation period (*p*<0.0001) and age of death (*p*=0.0008) but no differences were observed between the MM and VV and disease duration. Statistical significance was determined using a one-way ANOVA test followed by the Tukey’s multiple comparisons test. Statistical analysis of all data was performed using GraphPad Prism software.

**Table 4: PrP^res^ types found in each available CNS region of the iCJD cases examined.**

| **Study ID** | ***PRNP***  **codon 129** | **PrP^res^ type** | | | | | | | |
| --- | --- | --- | --- | --- | --- | --- | --- | --- | --- |
|  |  | **FC** | **TC** | **PC** | **OC** | **CbC** | **Th** | **SC** | **Consensus** |
| hGH-iCJD1 | MV | i+2 | - | - | - | - | - | - | i+2 |
| hGH-iCJD2 | VV | - | 2 | - | - | - | - | 2 | 2 |
| hGH-iCJD3 | MV | - | - | - | - | i+2 | - | - | i+2 |
| hGH-iCJD4 | VV | 2 | - | - | - | 2 | - | - | 2 |
| hGH-iCJD5 | VV | 2 | 2 | 2 | 2 | 2 | 2 | - | 2 |
| hGH-iCJD6 | VV | - | - | - | - | 2 | - | - | 2 |
| hGH-iCJD7 | MV | i+2 | - | - | - | i+2 | - | - | i+2 |
| hGH-iCJD8 | VV | 2 | - | - | - | 2 | - | - | 2 |
| hGH-iCJD9 | MV | i+2 | - | - | - | - | - | - | i+2 |
| hGH-iCJD10 | VV | 2 | - | - | - | 2 | - | - | 2 |
| hGH-iCJD11 | MV | i+2 | - | - | - | - | - | - | i+2 |
| hGH-iCJD12 | VV | 2 | 2 | - | - | 2 | - | - | 2 |
| hGH-iCJD13 | MV | i+2 | - | - | - | i+2 | - | - | i+2 |
| hGH-iCJD14 | MV | i+2 | - | - | - | i+2 | - | - | i+2 |
| hGH-iCJD15 | MV | i+2 | 2 | 2 | i+2 | i+2 | i+2 | - | i+2 |
| hGH-iCJD16 | MV | 2 | - | - | - | i+2 | - | - | i+2 |
| hGH-iCJD17 | MV | i+2 | - | - | - | i+2 | - | - | i+2 |
| hGH-iCJD18 | MV | i+2 | - | - | - | i+2 | - | - | i+2 |
| hGH-iCJD19 | MV | i+2 | i+2 | - | i+2 | i+2 | - | - | i+2 |
| hGH-iCJD20 | MM | i | i | i | i | i | i | - | i |
| hGH-iCJD21 | MM | - | 1 | - | - | - | - | - | 1 |
| hDM-iCJD1 | MM | 1 | - | - | - | - | - | - | 1 |
| hDM-iCJD2 | MM | 1 | 1 | 1 | 1 | 1 | 1 | - | 1 |
| hDM-iCJD3 | MM | 1 | - | 1 | - | 1 | - | - | 1 |

FC = frontal cortex, TC = temporal cortex, PC = parietal cortex, OC = occipital cortex, CbC = cerebellar cortex, Th = thalamus, SC = spinal cord, - = not tested

**Table 5: PrP^res^ types found in defined CNS regions of the 108 sCJD cases examined.**

| **Study ID** | ***PRNP* codon 129 genotype** | **PrP^res^ type** | | | | |
| --- | --- | --- | --- | --- | --- | --- |
|  |  | **TC** | **PC** | **OC** | **Th** | **Consensus** |
| sCJD1 | MM | 2 | 1 | 1 | 1 | 1+2 |
| sCJD2 | MM | 1+2 | 1+2 | 1+2 | 0 | 1+2 |
| sCJD3 | MM | 1 | 1 | 1 | 1 | 1 |
| sCJD4 | MM | 0 | 1 | 0 | 0 | 1 |
| sCJD5 | MM | 1 | 1 | 1 | 0 | 1 |
| sCJD6 | MV | 2 | 2 | 2 | 1+2 | 1+2 |
| sCJD7 | VV | 2 | 2 | 2 | 2 | 2 |
| sCJD8 | MM | 1 | 1 | 1 | 1 | 1 |
| sCJD9 | VV | 1 | 1 | 1 | 1 | 1 |
| sCJD10 | MM | 1 | 1 | 1 | 1 | 1 |
| sCJD11 | MM | 1 | 1 | 1 | 1 | 1 |
| sCJD12 | MV | 1+2 | 1+2 | 2 | 1+2 | 1+2 |
| sCJD13 | MM | 0 | 1 | 1 | 0 | 1 |
| sCJD14 | MM | 1 | 1 | 1 | 1 | 1 |
| sCJD15 | MM | 1 | 1 | 1 | 0 | 1 |
| sCJD16 | MM | 1 | 1 | 1 | 1 | 1 |
| sCJD17 | MM | 1+2 | 1 | 1 | 1 | 1+2 |
| sCJD18 | MM | 1 | 1 | 1 | 1 | 1 |
| sCJD19 | MM | 1 | 1 | 1 | 1 | 1 |
| sCJD20 | MM | 2 | 2 | 2 | 0 | 2 |
| sCJD21 | MM | 2 | 2 | 2 | 2 | 2 |
| sCJD22 | MM | 1 | 1 | 1 | 1 | 1 |
| sCJD23 | MV | i+2 | 2 | 2 | i+2 | i+2 |
| sCJD24 | VV | 2 | 2 | 2 | 2 | 2 |
| sCJD25 | MM | 1 | 1+2 | 1 | 1+2 | 1+2 |
| sCJD26 | MM | 1 | 1 | 1 | 1 | 1 |
| sCJD27 | MM | 1 | 1 | 1 | 1 | 1 |
| sCJD28 | MM | 1 | 1 | 1 | 0 | 1 |
| sCJD29 | MM | 1+2 | 2 | 2 | 2 | 1+2 |
| sCJD30 | MM | 0 | 2 | 2 | 1+2 | 1+2 |
| sCJD31 | MM | 1 | 1 | 1 | 1 | 1 |
| sCJD32 | VV | 2 | 2 | 2 | 2 | 2 |
| sCJD33 | MM | 1 | 1 | 1 | 1 | 1 |
| sCJD34 | MV | 1 | 1 | 1 | 1 | 1 |
| sCJD35 | MM | 1 | 1 | 1 | 1 | 1 |
| sCJD36 | VV | 2 | 2 | 2 | 2 | 2 |
| sCJD37 | MM | 1 | 1 | 1 | 0 | 1 |
| sCJD38 | VV | 2 | 2 | 2 | 2 | 2 |
| sCJD39 | MV | 2 | 2 | 2 | i+2 | i+2 |
| sCJD40 | VV | 2 | 2 | 2 | 2 | 2 |
| sCJD41 | MM | 1 | 1 | 1 | 1 | 1 |
| sCJD42 | MM | 1 | 1 | 1+2 | 1+2 | 1+2 |
| sCJD43 | MV | 2 | 2 | 2 | 2 | 2 |
| sCJD44 | MM | 1 | 1 | 1 | 1 | 1 |
| sCJD45 | MV | 1 | 1 | 1 | 1 | 1 |
| sCJD46 | MM | 1 | 1 | 1 | 1 | 1 |
| sCJD47 | MM | 1 | 1 | 1 | 1 | 1 |
| sCJD48 | MM | 1 | 1 | 1 | 0 | 1 |
| sCJD49 | MM | 1 | 1 | 1+2 | 1 | 1+2 |
| sCJD50 | MM | 1 | 1 | 1 | 1 | 1 |
| sCJD51 | MM | 1 | 1 | 1 | 1+2 | 1+2 |
| sCJD52 | MM | 1 | 1 | 1 | 1 | 1 |
| sCJD53 | MM | 1 | 1 | 1 | 1 | 1 |
| sCJD54 | MM | 1 | 1 | 1 | 1 | 1 |
| sCJD55 | MV | 2 | 2 | 2 | i+2 | i+2 |
| sCJD56 | MM | 1 | 1 | 1 | 1 | 1 |
| sCJD57 | MM | 1 | 1 | 1 | 1+2 | 1+2 |
| sCJD58 | MV | 2 | 1+2 | 1+2 | 1+2 | 1+2 |
| sCJD59 | MM | 1 | 1 | 1 | 0 | 1 |
| sCJD60 | MM | 1 | 1 | 1 | 1 | 1 |
| sCJD61 | MM | 1 | 1 | 1 | 1 | 1 |
| sCJD62 | MM | 1+2 | 1+2 | 1+2 | 1+2 | 1+2 |
| sCJD63 | VV | 2 | 2 | 2 | 2 | 2 |
| sCJD64 | MM | 1 | 1+2 | 1+2 | 1+2 | 1+2 |
| sCJD65 | MV | 0 | 0 | 0 | 1 | 1 |
| sCJD66 | MV | i+2 | i+2 | 2 | i+2 | i+2 |
| sCJD67 | MM | 2 | 2 | 0 | 1+2 | 1+2 |
| sCJD68 | MM | 2 | 2 | 2 | 2 | 2 |
| sCJD69 | MM | 1 | 1+2 | 1+2 | 1 | 1+2 |
| sCJD70 | MV | 1+2 | 1 | 1+2 | 1+2 | 1+2 |
| sCJD71 | MM | 1+2 | 1+2 | 1+2 | 1+2 | 1+2 |
| sCJD72 | MM | 1 | 1 | 1 | 1 | 1 |
| sCJD73 | MM | 1 | 1 | 1 | 1 | 1 |
| sCJD74 | MM | 1 | 1 | 1 | 1 | 1 |
| sCJD75 | MM | 1 | 1 | 1 | 1 | 1 |
| sCJD76 | MV | 2 | 2 | 2 | 2 | 2 |
| sCJD77 | VV | 2 | 2 | 2 | 2 | 2 |
| sCJD78 | VV | 2 | 2 | 2 | 2 | 2 |
| sCJD79 | VV | 2 | 2 | 2 | 2 | 2 |
| sCJD80 | MV | 2 | 2 | 2 | 2 | 2 |
| sCJD81 | MM | 1 | 1+2 | 1+2 | 1 | 1+2 |
| sCJD82 | VV | 0 | 2 | 2 | 2 | 2 |
| sCJD83 | VV | 0 | 0 | 0 | 2 | 2 |
| sCJD84 | MV | 0 | 0 | 0 | 2 | 2 |
| sCJD85 | MV | 1 | 1 | 1 | 0 | 1 |
| sCJD86 | MM | 1 | 1 | 1 | 1 | 1 |
| sCJD87 | VV | 2 | 2 | 2 | 2 | 2 |
| sCJD88 | MM | 1+2 | 1+2 | 1 | 1+2 | 1+2 |
| sCJD89 | MM | 1 | 1 | 1 | 1 | 1 |
| sCJD90 | MM | 1 | 1+2 | 1+2 | 1 | 1+2 |
| sCJD91 | MV | 1+2 | 1+2 | 1+2 | 1+2 | 1+2 |
| sCJD92 | MM | 1 | 1 | 1 | 1 | 1 |
| sCJD93 | MM | 1 | 1 | 1 | 1 | 1 |
| sCJD94 | MM | 1 | 1 | 1 | 0 | 1 |
| sCJD95 | MV | 2 | 2 | 2 | 2 | 2 |
| sCJD96 | VV | 1 | 1+2 | 0 | 1+2 | 1+2 |
| sCJD97 | MM | 1 | 1 | 1 | 1 | 1 |
| sCJD98 | MM | 1+2 | 1+2 | 1 | 1+2 | 1+2 |
| sCJD99 | VV | 2 | 2 | 0 | 2 | 2 |
| sCJD100 | MV | 2 | 2 | 2 | 0 | 2 |
| sCJD101 | MM | 1 | 1 | 0 | 1 | 1 |
| sCJD102 | MM | 1 | 1 | 1 | 1 | 1 |
| sCJD103 | MM | 1 | 1 | 1 | 1 | 1 |
| sCJD104 | MM | 1 | 1 | 0 | 0 | 1 |
| sCJD105 | MM | 1 | 1 | 1 | 1 | 1 |
| sCJD106 | MM | 1 | 1 | 1 | 1 | 1 |
| sCJD107 | MM | 2 | 1+2 | 1 | 1+2 | 1+2 |
| sCJD108 | MM | 1 | 1 | 1 | 1 | 1 |

TC = temporal cortex, PC = parietal cortex, OC = occipital cortex, Th = thalamus

**Table 6: Classification of the 108 sCJD cases according to PrP^res^ types found in the defined CNS regions and the *PRNP* codon 129 genotype.**

|  |  | **Numbers of sCJD cases as classified by genotype and PrP^res^ type** | | | | |
| --- | --- | --- | --- | --- | --- | --- |
| ***PRNP* codon 129 genotype** | | **PrP^res^ type 1** | **PrP^res^ type i** | **PrP^res^ type 1+2** | **PrP^res^ type i+2** | **PrP^res^ type 2** |
| MM | | 50 | 0 | 20 | 0 | 3 |
| MV | | 4 | 0 | 5 | 4 | 6 |
| VV | | 1 | 0 | 1 | 0 | 14 |

**Table 7: Histotype and PrP^res^ type of *PRNP* codon 129 MV sCJD cases**

| **Study ID** | *PRNP* codon 129 genotype | PrP^res^ type | | | | | Histotype |
| --- | --- | --- | --- | --- | --- | --- | --- |
|  |  | TC | PC | OC | Th | Consensus |  |
| sCJD6 | MV | 2 | 2 | 2 | 1+2 | 1+2 | MV2K+2C |
| sCJD12 | MV | 1+2 | 1+2 | 2 | 1+2 | 1+2 | MV2K+2C^a^ |
| sCJD23 | MV | i+2 | 2 | 2 | i+2 | i+2 | MV2K^a^ |
| sCJD39 | MV | 2 | 2 | 2 | i+2 | i+2 | MV2K+2C |
| sCJD55 | MV | 2 | 2 | 2 | i+2 | i+2 | MV2K |
| sCJD58 | MV | 2 | 1+2 | 1+2 | 1+2 | 1+2 | MV2K+2C^a^ |
| sCJD66 | MV | i+2 | i+2 | 2 | i+2 | i+2 | MV2K^a^ |
| sCJD70 | MV | 1+2 | 1 | 1+2 | 1+2 | 1+2 | MV1+(2C)^a^ |
| sCJD91 | MV | 1+2 | 1+2 | 1+2 | 1+2 | 1+2 | MV2K+(2C)^a^ |

^a^Histotype reported previously in Moore et al 2016 [22]
